# Boosting SIV-specific CD8^+^ T cell responses prior to ART interruption extends time to SIVmac239 rebound

**DOI:** 10.1172/JCI198294

**Published:** 2026-01-29

**Authors:** Were R. Omange, Benjamin D. Varco-Merth, Omo Fadeyi, Alejandra Marenco, Hiroshi Takata, Derick M. Duell, William D. Goodwin, Paula Armitage, Christine M. Fennessey, Emek Kose, Taina T. Immonen, Ewelina Kosmider, William J. Bosche, Randy Fast, Chris Homick, Kelli Oswald, Rebecca Shoemaker, Rachele Bochart, Rhonda MacAllister, Caralyn S. Labriola, Jeremy V. Smedley, Michael K. Axthelm, Paul T. Edlefsen, Brandon F. Keele, Jeffrey D. Lifson, Janina Gergen, Benjamin Petsch, Susanne Rauch, Louis J. Picker, Afam A. Okoye

**Affiliations:** 1 Vaccine and Gene Therapy Institute and; 2 Oregon National Primate Research Center, Oregon Health & Science University, Beaverton, Oregon, USA.; 3 AIDS and Cancer Virus Program, Frederick National Laboratory for Cancer Research, Frederick, Maryland, USA.; 4 Fred Hutchinson Cancer Center, Seattle, Washington, USA.; 5 CureVac SE, Tübingen, Germany.

**Keywords:** AIDS/HIV, Immunology, AIDS vaccine, Adaptive immunity

## Abstract

HIV/SIV-specific CD8^+^ T cell responses are typically unable to control viral rebound following antiretroviral therapy (ART) interruption (ATI). To investigate whether enhancing the magnitude and activation of SIV-specific CD8^+^ T cells at the time of ATI can improve the immune interception of reactivating SIV infections, we vaccinated SIVmac239-infected rhesus macaques (RMs) on ART, boosting immediately prior to ATI, with a nucleoside-unmodified mRNA vaccine expressing SIVmac239 Gag (mRNA/SIVgag) alone or in combination with Nef (mRNA/SIVnef) and Pol (mRNA/SIVpol). The mRNA/SIVgag vaccine was effective in boosting Gag-specific CD8^+^ T cells in blood and lymphoid tissues. Following ATI, the mRNA/SIVgag vaccine group showed a significant delay in time to measurable viral rebound compared with controls and manifested lower plasma viral loads (PVLs) for up to 6 weeks after rebound. Similarly, RMs that received mRNA/SIVgag, mRNA/SIVnef, and mRNA/SIVpol also manifested a delay in SIV rebound compared with controls, suggesting that boosting SIV-specific CD8^+^ T cells during ATI can enhance early immune targeting of reactivating SIV infections. However, viral control was not sustained long term as PVLs were similar across vaccinees and controls by 24 weeks after rebound, highlighting the need for adjunctive therapies to improve the durability of virologic control elicited by CD8^+^ T cell–targeting vaccines.

## Abstract 2

A therapeutic vaccine strategy that boost virus-speciifc CD8+ T cells during ART interruption, delays viral rebound and promotes early control of viral replication.

## Graphical abstract

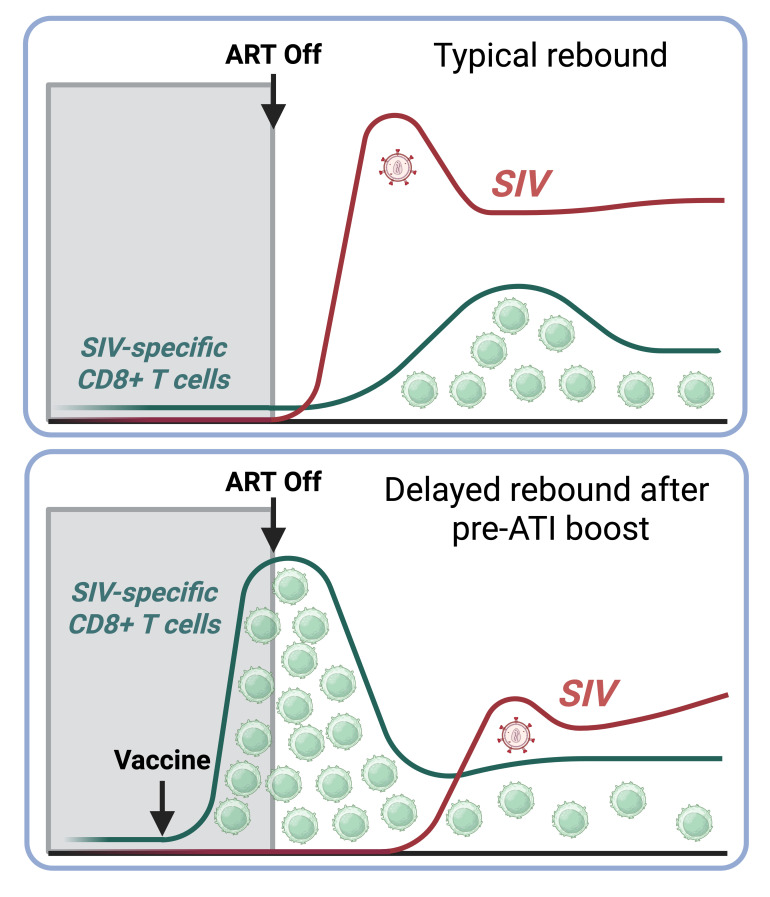

## Introduction

CD8^+^ T cells play a crucial role in the adaptive immune response to HIV infection, with CD8^+^ T cell immunity implicated in many examples of long-term, spontaneous, and vaccine-associated immune control of HIV and SIV infection (1–5). However, in the majority of people living with HIV (PWH), naturally occurring CD8^+^ T cell responses are insufficient to halt virus replication in the absence of antiretroviral therapy (ART) (6). As a result, lifelong ART is necessary to suppress ongoing virus replication and prevent disease progression. Despite its effectiveness, prolonged ART carries significant health risks for PWH and imposes a significant economic burden on society (7). Additionally, the stigma associated with treatment for HIV infection remains a global concern. Therefore, there is a need to develop novel therapeutic interventions capable of more definitive treatment of HIV infection, either through eradication of the virus or, perhaps more feasibly, through a treatment intervention where molecular or other evidence of infection may persist after ART cessation but detectable viral replication, disease progression, and viral transmission are fully and durably abrogated (8, 9).

Given the potential of optimal CD8^+^ T cell responses to suppress HIV infection, a key question is whether HIV-specific CD8^+^ T cells can be more generally harnessed in PWH to fully control viral replication after ART withdrawal. Previously, to gain insights into the functional role and potential of naturally induced CD8^+^ T cell responses during viral rebound, we administered a CD8β-depleting mAb to SIVmac239-infected rhesus macaques (RMs) during ART interruption (ATI) (10). CD8β-depleting mAbs were used to specifically eliminate classical CD8αβ^+^ cells, while other CD8α^+^ subsets, such as NK cells and CD4^+^ effector memory T cells, remained unaffected. Remarkably, the depletion of CD8^+^ T cells had no measurable impact on the time to detectable plasma viremia, indicating that SIV-specific CD8^+^ T cell responses elicited during acute SIV infection and maintained on ART were limited in their ability to intercept and control reactivating reservoirs and/or early viral spread. However, peak post-ART plasma viral load (PVL) set points were approximately 2 logs higher in CD8^+^ T cell–depleted RMs compared with controls, suggesting CD8^+^ T cell responses can exert measurable virologic control, but only after viral amplification and systemic spread provide sufficient levels of antigen to drive anamnestic expansion of these antiviral T cell responses. Since this effector CD8^+^ T cell activity appears to occur too late to delay initial rebound, it remains undetermined whether immune interventions that enhance the magnitude, breadth, and/or functional activity of virus-specific CD8^+^ T cell responses during the initial stages of ATI would be able to alter the dynamic mismatch between a lagging immune response and a rapidly amplifying virus population to prevent or delay rebound.

Therapeutic vaccines have been extensively studied as a means to control post-ART rebound, with the goal of these vaccines traditionally being the boosting of the magnitude of preexisting virus-specific CD8^+^ T cells and the generation of entirely new responses to epitopes that were not targeted by naturally developing responses to infection (11–14). To date, these studies have shown limited efficacy in delaying or preventing rebound, even with highly immunogenic vaccines (15–20), suggesting that merely increasing steady-state frequencies of preexisting or newly induced epitope-specific responses during ART is insufficient to overcome the temporal mismatch between delayed immune responses and virus rebound after ATI. Importantly, these studies have administered the vaccine during ART with the goal of enhancing CD8^+^ T cell responses on ART, while still relying on the post-ATI viral rebound itself to trigger the anamnestic response. Thus, even the vaccine-enhanced anamnestic response would intercept the infection relatively late in the rebound process, after extensive viral replication and spread. We hypothesized that a strategically timed boost immediately prior to ATI could initiate the anamnestic response prior to rebound, accelerating the immune intercept of the rebounding infection, possibly increasing efficacy.

Therefore, the aim of this study was to assess whether a therapeutic vaccination strategy that induces high frequencies of potent, systemically distributed, SIV-specific CD8^+^ T cell responses during ART, with pre-ATI boosting timed to provide abundant, activated, SIV-specific CD8^+^ T cell responses in the earliest phases of post-ART rebound, could modify the rate and extent of viral recrudescence following ATI. To test this, SIVmac239-infected RMs on ART were immunized with nucleoside unmodified mRNA vaccines based on the RNActive platform (CureVac SE) (21, 22), which were engineered to express either SIV Gag alone or SIV Gag in combination with Nef and Pol, or for a negative control, rabies antigen. The SIV or control vaccines were administered 5 times on ART, with the last dose given 2 weeks before ATI to boost responses just prior to rebound. We found that the active vaccine dramatically accelerated the SIV-specific CD8^+^ T cell response after ATI, substantially delaying rebound and reducing early viral replication compared with RMs given the control vaccine. However, the protection observed in the RMs given the active vaccine waned after 6 weeks, and long-term viral loads were similar to the control group, indicting that early CD8^+^ T cell interception alone is insufficient for durable post-ART viral control.

## Results

### Study design.

**Figure 1:**
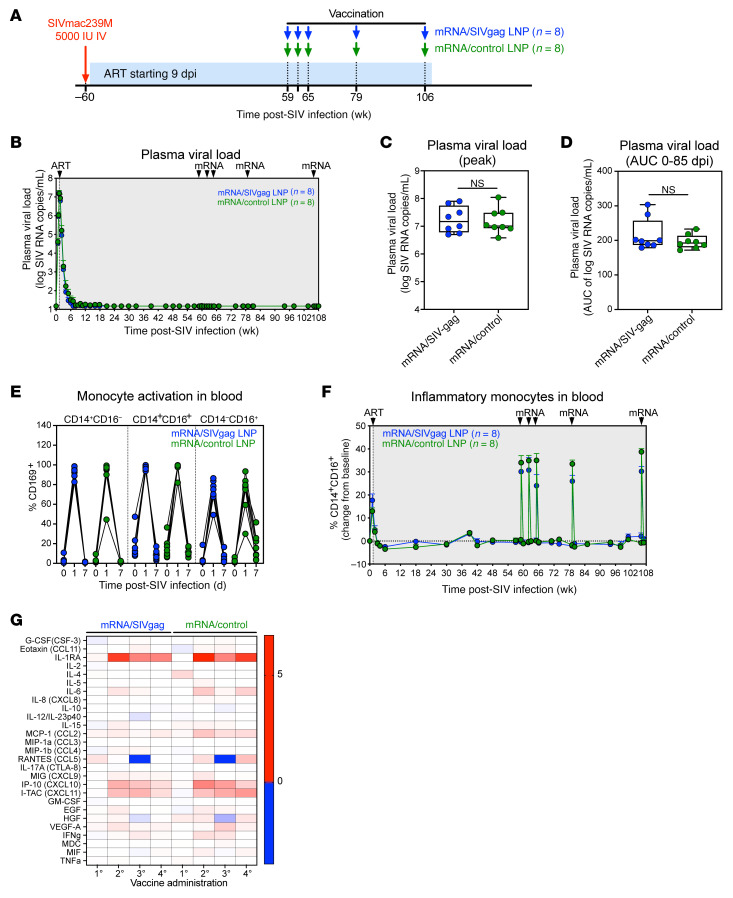
mRNA/SIVgag vaccination increases monocyte activation in SIV-infected RM on ART. (**A**) Schematic representation of the study protocol, including SIVmac239 infection, initiation of ART at 9 dpi, and vaccination with either mRNA/SIVgag or mRNA/control vaccines at 59, 62, 65, 79, and 106 wpi. (**B**) Mean (+SEM) PVL profiles of RMs in the mRNA/SIVgag vaccine group (*n* = 8) compared with mRNA/control group (*n* = 8), prior to ART discontinuation. (**C** and **D**) Comparison of peak PVL (**C**) and AUC of PVL (**D**) from 0 to 85 dpi between the 2 vaccine groups. Box-and-whisker plots show jittered points, a box from first to third quartiles (IQR), and a line at the median, with whiskers down to the minimum and up to the maximum value. (**E**) CD169 expression on the designated monocyte subsets in blood at 0, 1, and 7 days after vaccination. (**F**) Mean (+SEM) frequencies of CD14^+^CD16^+^ monocytes as a fraction of CD3^–^CD20^–^CD8^–^HLA-DR^+^ lymphocytes in blood of RMs in the mRNA/SIVgag vaccine group (*n* = 8) versus the mRNA/control group (*n* = 8), prior to ART discontinuation. (**G**) Heatmap showing change in plasma cytokine/chemokine levels in each vaccine group between 0 and 24 hours after vaccination. Statistical significance between groups was determined by Wilcoxon’s rank-sum test; *P* values ≤ 0.05 are indicated.

The goals of this study were 2-fold: (a) to evaluate whether increasing frequencies and functional activity of SIV-specific CD8^+^ T cells during ART, including with a boost just prior to ATI, can delay the rate and extent of SIVmac239 rebound, which would provide evidence that CD8^+^ T cells can intercept foci of reactivating, persistent, tissue-level infections, and (b) to determine whether proactively inducing the CD8^+^ T cell response to precede rebounding viremia can support durable post-ART viral control. To test this, we initially constructed a nucleoside unmodified mRNA vaccine to express full-length SIVmac239 Gag (mRNA/SIVgag) and encapsulated in lipid nanoparticles (LNPs), with the aim of specifically boosting Gag-specific CD8^+^ T cell responses, in part due to the important role of Gag responses in promoting viral control (23), comparing this Gag-expressing vaccine to a similar construct that expressed an irrelevant antigen (rabies virus glycoprotein) (24). The study included 16 RMs, which were intravenously inoculated with 5,000 IU of the barcoded SIVmac239M viral stock, followed by ART consisting of tenofovir disoproxil fumarate, emtricitabine, and dolutegravir starting 9 days postinfection (dpi) (25) (Figure 1A). After stable ART suppression for approximately 58 weeks, the RMs were divided into 2 balanced groups based on age, sex, and MHC-I alleles (Supplemental Table 1; supplemental material available online with this article; https://doi.org/10.1172/JCI198294DS1). These groups received 5 intramuscular injections of either the mRNA/SIVgag (*n* = 8) or mRNA/control vaccines (*n* = 8) at 59, 62, 65, 79, and 106 weeks postinfection (wpi), while receiving continued ART, with the final immunization occurring approximately 2 weeks prior to ATI (~108 wpi).

In response to ART, PVL declined to below 15 SIV RNA copies/mL in most RMs by 8 wpi (Figure 1B). Peak PVL and viral burden as measured by the AUC of PVL between 0 and 12 wpi were not statistically different between mRNA/SIVgag-vaccinated RMs and control group (Figure 1, C and D). Each mRNA vaccination was associated with a substantial, yet transient, increase in the activation marker CD169 on all monocyte subsets in blood, including CD14^+^CD16^–^, CD14^+^CD16^+^, and CD14^–^CD16^+^ monocytes (Figure 1E and Supplemental Figure 1A). Longitudinal analyses also revealed increases in the proinflammatory CD14^+^CD16^+^ monocyte subset in blood following each vaccination (Figure 1F and Supplemental Figure 1B), suggesting that RNActive vaccines rapidly, but transiently, elevate levels of inflammation. Notably, classical CD14^+^CD16^–^ monocytes transiently decreased while nonclassical CD14^–^CD16^+^ monocytes were generally stable after vaccination (Supplemental Figure 1, C and D). Finally, analysis of soluble immune mediators in plasma 24 hours after each vaccination revealed increases in proinflammatory cytokines such as IL-1RA, IP-10, I-TAC, IL-6, and MCP-1 in both vaccine groups (Figure 1G). However, no measurable increase in plasma viral blips above 15 SIV RNA copies/mL was observed in either group after vaccination (Figure 1B). Collectively, these data demonstrate that RNActive vaccines are safe, transiently increase innate immune activation, and do not induce measurable virus reactivation in SIV-infected RMs on ART.

### mRNA/SIVgag vaccination increases Gag-specific CD8^+^ T cells in SIV-infected RM on ART.

**Figure 2:**
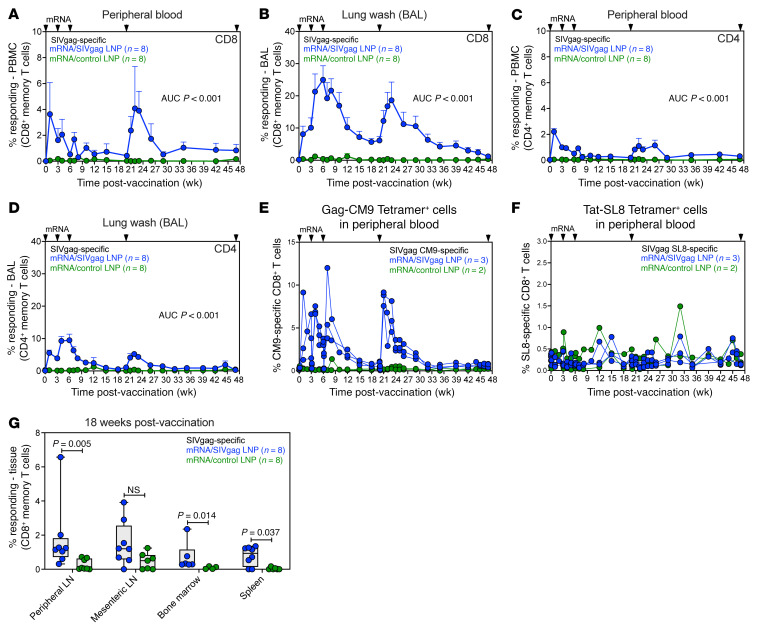
mRNA/SIVgag vaccination induces robust CD8^+^ T cell responses in SIV-infected RMs on ART. (**A**–**D**) Comparison of SIV Gag–specific T cell responses in RMs from the mRNA/SIVgag group versus the mRNA/control group: CD8^+^ T cell responses in blood (**A**) and BAL (**B**), and CD4^+^ T cell responses in blood (**C**) and BAL (**D**). Data represent mean frequencies (*n* = 8, +SEM). (**E** and **F**) Frequencies of peripheral blood CD8^+^ T cells specific for SIV Gag-CM9 (**E**) and Tat-SL8 (**F**), as determined by MHC-I multimer staining as described in Methods. (**G**) Comparison of SIV Gag–specific CD8^+^ T cell responses in PLNs, MLNs, BM, and spleen. Each data point represents an individual RM. Box-and -whisker plots show jittered points, a box from first to third quartiles (IQR), and a line at the median, with whiskers down to the minimum and up to the maximum value. Statistical significance between groups was determined by Wilcoxon’s rank-sum test; *P* values ≤ 0.05 are indicated.

After vaccination with the mRNA/SIVgag and mRNA/control vaccines, flow cytometric intracellular cytokine staining (ICS) was used to measure the vaccine-elicited CD8^+^ T cell response to consecutive, overlapping Gag 15-mer peptide pools (Supplemental Figure 2). Longitudinal analysis revealed that SIV-specific CD4^+^ and CD8^+^ T cell responses in blood and bronchoalveolar lavage (BAL) fluid (representing an immune effector site) were significantly higher in mRNA/SIVgag-vaccinated RMs compared with mRNA/control-vaccinated animals (*P* < 0.001 for all comparisons). Gag-specific CD8^+^ T cell responses in the mRNA/SIVgag group peaked at a mean (+SEM) of 4.1% (+3.2%) and 25% (+4.4%) in blood and BAL, respectively. In comparison, Gag-specific CD4^+^ T cell responses peaked at a mean (+SEM) of 2.2% (+0.2%) and 9.5% (+1.9%) in blood and BAL, respectively (Figure 2, A–D).

By design, a subset of 3 RMs in the mRNA/SIVgag vaccine group and 2 RMs in the mRNA/control group expressed the MHC-I allele *Mamu* A*01, which allowed for the quantification of canonical immunodominant Gag-CM9 and Tat-SL8 epitope-specific CD8^+^ T cell responses by flow cytometric MHC-I multimer staining (Supplemental Figure 3A). Since the mRNA/SIVgag construct encodes for Gag alone, comparisons could be made between native Tat-SL8 epitope-specific responses (Tat is not included in the vaccine) and Gag-CM9 epitope-specific responses, which should be boosted following vaccination. As shown in Figure 2E, there was a marked increase in the frequencies of Gag-CM9^+^CD8^+^ T cells in the blood in response to mRNA/SIVgag vaccination. In contrast, frequencies of Tat-SL8^+^ T cells did not show substantial changes following vaccination (Figure 2F).

Analyses of CD8^+^ T cell responses in peripheral lymph nodes (PLNs), mesenteric lymph nodes (MLNs), spleen, and bone marrow (BM) by week 18 after first vaccination also revealed higher frequencies of Gag-specific CD8^+^ T cell responses in the mRNA/SIVgag group compared with the mRNA/control group (Figure 2G). This was further confirmed by Gag-CM9^+^ MHC-I multimer staining across several lymphoid tissues in the *Mamu* A*01^+^ RMs, which revealed higher frequencies of Gag-specific CD8^+^ T cells in PLNs, MLNs, spleen, BM, BAL, liver, small intestine, and rectum in mRNA/SIVgag-vaccinated RMs compared with controls (Supplemental Figure 3B). Collectively, these data demonstrate that the mRNA/SIVgag vaccine is highly immunogenic in SIV-infected RMs on ART and induces high frequencies of SIV-specific CD8^+^ T cells in both blood and lymphoid tissue compartments.

### mRNA/SIVgag vaccination increases Gag-specific CD8^+^ T cells during ATI.

**Figure 3:**
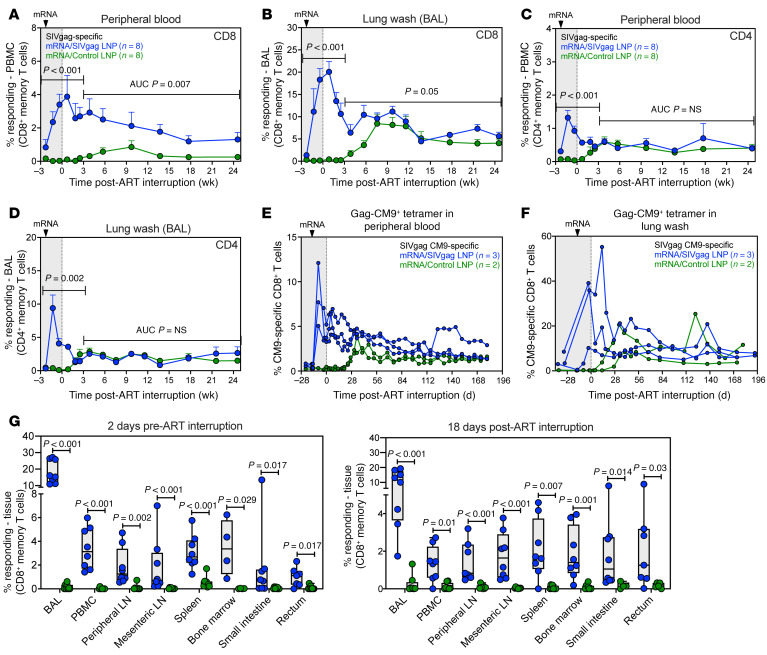
A booster mRNA/SIVgag vaccination just prior to ART cessation enhances CD8^+^ T cell responses during ATI. (**A**–**D**) Comparison of SIV Gag–specific CD8^+^ T cell responses in RMs from the mRNA/SIVgag group versus the mRNA/control group: CD8^+^ T cell responses in blood (**A**) and BAL (**B**), and CD4^+^ T cell responses in blood (**C**) and BAL (**D**). Data represent mean frequencies (*n* = 8, + SEM). (**E** and **F**) Frequencies of peripheral blood CD8^+^ T cells specific for SIV Gag-CM9 in blood (**E**) and BAL (**F**), as determined by MHC-I multimer staining as described in Methods. (**G**) Comparison of SIV Gag–specific CD8^+^ T cell responses in lung wash (BAL), blood, PLNs, MLNs, spleen, BM, small intestine, and rectum at 2 time points: 2 days prior to and 18 days after ATI. Each data point represents an individual RM. Box-and-whisker plots show jittered points, a box from first to third quartiles (IQR), and a line at the median, with whiskers down to the minimum and up to the maximum value. Statistical significance between groups was determined by Wilcoxon’s rank-sum test; *P* values ≤ 0.05 are indicated.

To determine whether increasing the frequencies of SIV-specific CD8^+^ T cells at the time of ART discontinuation can enhance the interception of viral reactivation and rebound, a final boost mRNA/SIVgag vaccination was administered approximately 2 weeks prior to ATI. As expected, there was a marked increase in Gag-specific CD8^+^ T cells in mRNA/SIVgag-vaccinated RMs that peaked at a mean (+SEM) of 3.9% (+1.3%) and 21% (+2.3%) of CD8^+^ memory T cells in blood and BAL, respectively, by day 10 after ATI (Figure 3, A and B). Of note, increased frequencies of Gag-specific CD4^+^ T cell responses were also observed, although they peaked lower at a mean (+SEM) of 1.3% (+0.2%) and 9.4% (+2%) CD4^+^ memory T cells in blood and BAL, respectively (Figure 3, C and D). However, Gag-specific CD4^+^ T cell responses in both tissues rapidly declined after vaccination such that by 3 weeks after ART, there was no statistical difference between vaccinated RMs and controls. Flow cytometric tetramer staining of epitope-specific responses in *Mamu* A*01^+^ RMs also revealed that the final boost mRNA/SIVgag vaccination increased frequencies of Gag-CM9^+^CD8^+^ T cells in blood and BAL during ATI (Figure 3, E and F). In contrast, mRNA/control-vaccinated RMs exhibited a gradual increase in Gag-specific CD8^+^ T cells in blood and BAL starting approximately 3 weeks after ATI. A similar expansion of Tat-SL8^+^CD8^+^ T cells was also observed in mRNA/control-vaccinated RMs after ART discontinuation (Supplemental Figure 4, A and B). Both of these responses were indicative of antigen-driven T cell expansion in response to rebounding viremia.

To confirm our objective of systemically increasing Gag-specific CD8^+^ T cells at the time of ATI, we measured the mRNA vaccine–elicited CD8^+^ T cell response in several lymphoid tissues, including PLNs, MLNs, spleen, BM, liver, small intestine, and rectum, using ICS. As shown in Figure 3G, mRNA/gag-vaccinated RMs demonstrated statistically significantly higher frequencies of Gag-specific CD8^+^ T cells in all tissues sampled 2 days before and 18 days after ATI. These data demonstrate that boosting with mRNA/SIVgag vaccination just prior to ATI can substantially enhance CD8^+^ T cell responses across multiple lymphoid tissue compartments, including mucosal tissue sites that likely contribute to early post-ART viral spread (26).

### mRNA/SIVgag vaccination delays time to SIV rebound and enhances early post-ART viral control.

**Figure 4:**
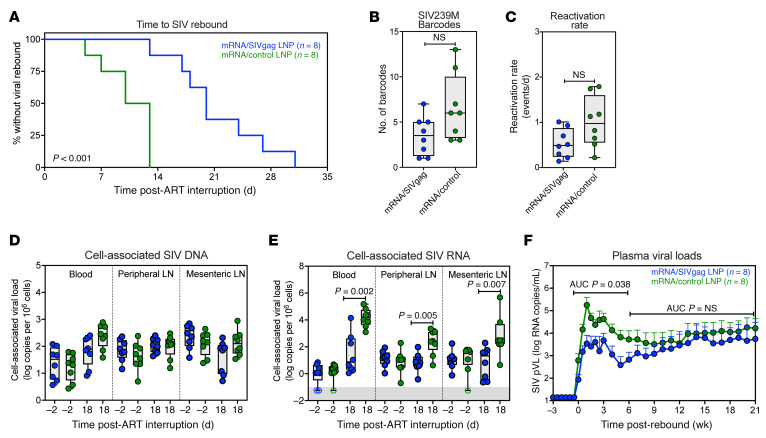
CD8^+^ T cell responses induced by the mRNA/SIVgag vaccine delay viral rebound and restrict early viral spread. (**A**) Kaplan-Meier analysis of SIV rebound kinetics in RMs from the mRNA/SIVgag group (*n* = 8) versus the mRNA/control group (*n* = 8). (**B**) Comparison of SIVmac239M proportions and (**C**) viral barcode clonal reactivation rates in plasma, as determined by high-throughput sequencing following ATI, between the 2 vaccine groups. (**D** and **E**) Comparison of SIV DNA (**D**) and SIV RNA (**E**) levels in blood, PLNs, and MLNs (copies per 10^6^ cell equivalents) at 2 days prior to and 18 days after ATI. Threshold sensitivity varied as a function of the number of cells available for analysis; for graphing, consistency values are plotted as unfilled symbols with a common nominal sensitivity threshold of 0.06 copies of SIV RNA per 10^6^ cell equivalents, indicated by the gray area. (**F**) Mean (+SEM) PVL profiles of RMs in the mRNA/SIVgag vaccine group (*n* = 8) versus the mRNA/control group (*n* = 8) normalized to rebound. Box-and-whisker plots show jittered points, a box from first to third quartiles (IQR), and a line at the median, with whiskers down to the minimum and up to the maximum value. Statistical significance between groups was determined by Wilcoxon’s rank-sum test; *P* values ≤ 0.05 are indicated.

We next determined whether these enhanced T cell responses manifested an impact on post-ART viral dynamics. Notably, we observed a substantial delay in the time to measurable rebound viremia in mRNA/SIVgag-vaccinated RMs compared with control vaccinated RMs (Figure 4A). Specifically, viral rebound (defined as the first of 3 consecutive off ART time points > 15 SIV RNA copies/mL) occurred at a median of 20 days after ART in mRNA/SIVgag-vaccinated RMs compared with a median of 11.5 days in controls (*P* < 0.001, log-rank test). Additionally, fewer SIVmac239M viral barcode clonotypes contributed to post-ART viremia early after ART discontinuation in mRNA/SIVgag-vaccinated RMs compared with controls, though this difference did not reach statistical significance (*P* = 0.153) (Figure 4B and Supplemental Figure 5). We also determined the growth rate of total plasma SIV RNA and used the proportional representation of individual SIVmac239M barcode clonotypes in relation to total rebound viremia at each time point to estimate the viral reactivation rate in individual RMs, as previously described (27, 28). The average rates of viral barcode clone reactivation were lower in mRNA/gag-vaccinated RMs compared with controls, although this difference also did not reach statistical significance (*P* = 0.074) (Figure 4C).

The delay in viral rebound and the marginal reductions in both the number of SIVmac239M variants contributing to rebound and their calculated reactivation rates suggest that SIV-specific CD8^+^ T cell responses induced by mRNA/SIVgag vaccination can provide early restriction of post-ART viral spread. To explore this further, we compared levels of cell-associated SIV DNA and SIV RNA in blood, PLNs, and MLNs between vaccine groups, both 2 days before and 18 days after ART discontinuation. While SIV DNA levels were not significantly different between mRNA/SIVgag-vaccinated RMs and controls before ATI and 18 days after ART (Figure 4D), there was a significant increase in cell-associated SIV RNA levels in PBMCs (*P* = 0.002), PLNs (*P* = 0.005), and MLNs (*P* = 0.007) of mRNA/control-vaccinated RMs by day 18 after ART (Figure 4E), consistent with CD8^+^ T cell responses in mRNA/SIVgag-vaccinated RMs limiting levels of SIV RNA in tissues after 18 days of ART discontinuation.

Once rebound occurred, there was an early restriction in post-ART viremia, as measured by the AUC of PVL between –1 and 6 weeks following rebound (*P* = 0.038) in the mRNA/SIVgag group (Figure 4F), suggesting that SIV-specific CD8^+^ T cell responses induced by mRNA/SIVgag were effective at limiting levels of SIV replication early after ATI. However, this was not sufficient to induce durable post-ART viral control as PVL gradually increased in the mRNA/SIVgag vaccine group, such that by 6–21 weeks after ART, levels of SIV RNA in plasma were not significantly different between the 2 vaccine groups.

**Figure 5:**
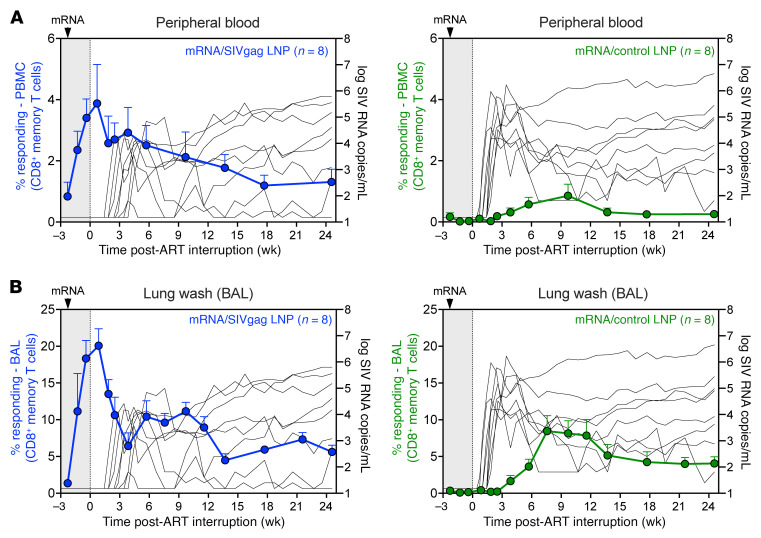
SIV rebound in mRNA/SIVgag-vaccinated RMs coincided with a decline in Gag-specific CD8^+^ T cells. (**A**) Overlay of the average frequency (*n* = 8, +SEM) of SIV Gag–specific CD8^+^ T cell responses in blood with individual SIV PVL profiles of RMs in the mRNA/SIVgag group (left) and the mRNA/control group (right). (**B**) A similar overlay is used for the responses in BAL.

Collectively, these data demonstrate the potency of CD8^+^ T cells against the initial stages of post-ART SIV rebound. As shown in Figure 5A, SIV rebound was restricted in the mRNA/gag vaccine group as SIV-specific CD8^+^ T cell responses peaked after vaccination. However, as these responses declined from peak in both blood and BAL, mRNA/SIVgag-vaccinated RMs began to manifest increases in plasma viremia. In contrast, SIV rebound in the mRNA/control group preceded the expansion of SIV-specific CD8^+^ T cell responses (Figure 5B). However, despite evidence of early virologic control, mRNA/SIVgag vaccination alone was not sufficient to facilitate durable virologic control. Full genome sequencing of plasma SIV from a subset of mRNA/SIVgag-vaccinated RMs with high post-ART viremia revealed an accumulation of point mutations across several epitopes, consistent with immune pressure (Supplemental Figure 6). However, while 2 RMs exhibited escape mutations in the putative Gag_241-249_ CD8^+^ T cell epitope (29), the other 2 showed no fixed mutations in Gag. These findings suggest that the lack of durable control in mRNA/SIVgag-vaccinated RMs was not invariably due to escape from Gag-specific CD8^+^ T cell responses induced by vaccination.

### mRNA/SIVgag/nef/pol vaccination also delays time to SIV rebound but results in limited enhancement of post-ART viral control.

**Figure 6:**
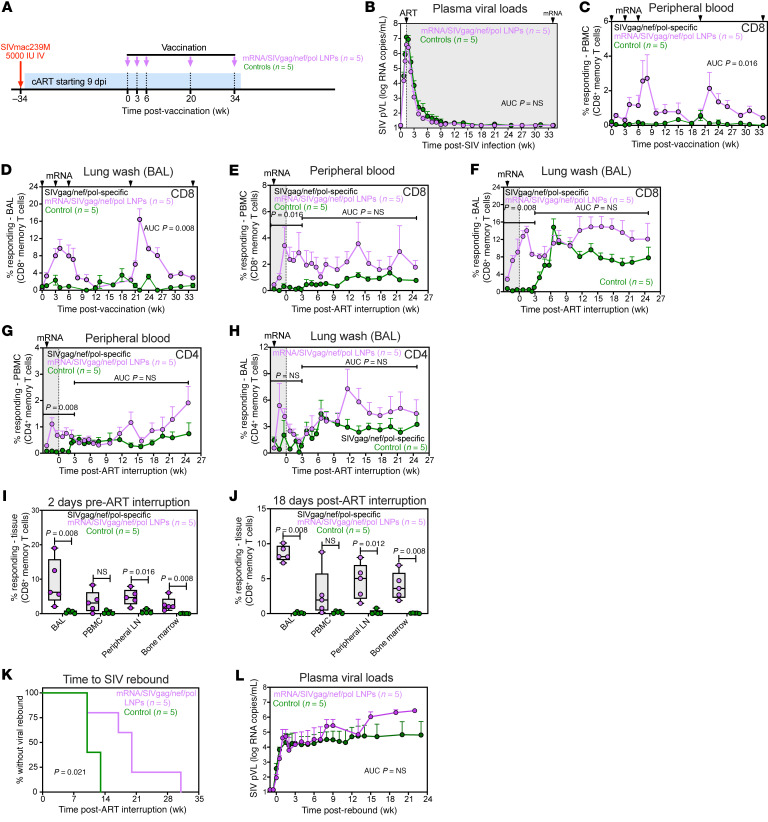
Combining mRNA/SIVgag with Nef and Pol vaccines delays SIV rebound but does not improve early post-ART viral control. (**A**) Schematic representation of the study protocol including SIVmac239 infection, initiation of ART at 9 dpi, mRNA/SIVgag/nef/pol vaccines, or no vaccination at 239, 260, 281, 379, and 476 dpi. (**B**) Mean (+SEM) PVL profiles of RMs in the mRNA/SIVgag/nef/pol group (*n* = 5) versus untreated controls (*n* = 5) prior to ATI. (**C** and **D**) Comparison of total SIV-specific CD8^+^ T cell responses (sum of Gag, Nef, and Pol) in blood (**C**) and BAL (**D**) during ART. (**E** and **F**) Total SIV-specific CD8^+^ T cell responses in blood (**E**) and BAL (**F**). (**G** and **H**) Total SIV-specific CD4^+^ T cell responses in blood (**G**) and BAL (**H**) during ATI. Data represent mean frequencies (*n* = 5, +SEM). (**I** and **J**) Comparison of SIV Gag–specific CD8^+^ T cell responses in BAL, blood, PLNs, and BM at 2 days prior to (**I**) and 18 days after (**J**) ATI. Each data point represents an individual RM. Box-and-whisker plots show jittered points, a box from first to third quartiles (IQR), and a line at the median, with whiskers down to the minimum and up to the maximum value. (**K**) Kaplan-Meier analysis of SIV rebound kinetics in the mRNA/SIVgag/nef/pol group versus controls (*n* = 5 per group). (**L**) Mean (+SEM) PVL profiles of RMs in both groups following ATI. Statistical significance between groups was determined by Wilcoxon’s rank-sum test; *P* values ≤ 0.05 are indicated.

We next determined whether post-ART viral control could be enhanced by broadening the vaccine-induced CD8^+^ T cell response to include Nef and Pol, in addition to Gag. To test this, we constructed 2 additional vaccines that expressed full-length SIVmac239 Nef (mRNA/SIVnef) and Pol (mRNA/SIVpol). The study involved 10 RMs, which were intravenously inoculated with 5,000 IU of SIVmac239M, followed by ART consisting of tenofovir disoproxil fumarate, emtricitabine, and dolutegravir starting at 9 dpi (Figure 6A). After stable ART suppression for approximately 33 weeks, the RMs were divided into 2 groups (Supplemental Table 2). These groups received 5 intramuscular injections of mRNA/SIVgag + mRNA/SIVnef + mRNA/SIVpol (mRNA/SIVgag/nef/pol; *n* = 5) at 100 μg each (300 μg total) or no treatment (*n* = 5) at 34, 37, 40, 54, and 68 wpi, with the final immunization occurring 2 weeks prior to ART discontinuation at 70 wpi.

In response to ART, PVL declined to below 15 SIV RNA copies/mL by 9 wpi (Figure 6B). After vaccination with the mRNA/SIVgag/nef/pol vectors, flow cytometric ICS was used to measure the vaccine-elicited CD8^+^ T cell response to Gag, Nef, and Pol peptide pools. Longitudinal analysis revealed that total SIV-specific CD8^+^ T cell responses (summed across Gag, Nef, and Pol) were significantly higher in blood (*P* = 0.016) and BAL (*P* = 0.008) of mRNA/SIVgag/nef/pol-vaccinated RMs compared with unvaccinated controls (Figure 6, C and D). Notably, CD8^+^ T cell responses following vaccination were higher for Gag compared with Nef or Pol. Gag-specific CD8^+^ T cells peaked at a mean (+SEM) of 2.1% (+0.9%) and 10.2% (+1.8%) in blood and BAL, respectively, compared with a mean (+SEM) of 0.4% (+0.1%) and 5.5% (+1.4%) for Nef and 0.5% (+0.5%) and 2% (+0.2%) for Pol in blood and BAL, respectively (Supplemental Figure 7). A final boost mRNA/SIVgag/nef/pol vaccination was administered approximately 2 weeks prior to ART discontinuation, and as anticipated, there was a significant increase in total SIV-specific CD8^+^ T cells in both blood (*P* = 0.016) and BAL (*P* = 0.008) between vaccinated RMs and controls through the first 3 weeks of ATI (Figure 6, E and F). Gag-specific CD4^+^ T cell responses were also significantly higher in blood (*P* = 0.008) but not BAL through the same period (Figure 6, G and H). We also compared vaccine-elicited CD8^+^ T cell response in tissues both 2 days before and 18 days after ATI and observed significantly higher frequencies of SIV-specific CD8^+^ T cells in BAL (2 days before ATI, *P* = 0.008; 18 days after ATI, *P* = 0.008), PLNs (2 days before ATI, *P* = 0.016; 18 days after ATI, *P* = 0.012), and BM (2 days before ATI, *P* = 0.008; 18 days after ATI, *P* = 0.008) (Figure 6, I and J).

After 70 weeks of ART administration and 2 weeks following the final mRNA/SIVgag/nef/pol vaccination, ART was discontinued, and viral rebound dynamics were assessed. Similar to the Gag-only vaccine, a significant delay in the time to measurable rebound viremia was observed in mRNA/SIVgag/nef/pol-vaccinated RMs compared with controls. Specifically, viral rebound occurred at a median of 20 days after ART in mRNA/SIVgag/nef/pol-vaccinated RMs compared with a median of 10 days in controls (*P* = 0.021, log-rank test) (Figure 6K). However, levels of viremia were not significantly different between vaccinated RMs and controls (Figure 6L). Collectively, these data suggest that broadening the CD8^+^ T cell response at the time of ATI by adding Pol and Nef gene inserts into the mRNA-based vaccination protocol, while sufficient to extend time to viral rebound relative to controls, did not enhance the durability of post-ART viral control.

### Addition of mRNA/SIVnef and mRNA/SIVpol diminished SIV-Gag–specific CD8^+^ T cell responses compared with responses elicited by mRNA/SIVgag alone.

**Figure 7:**
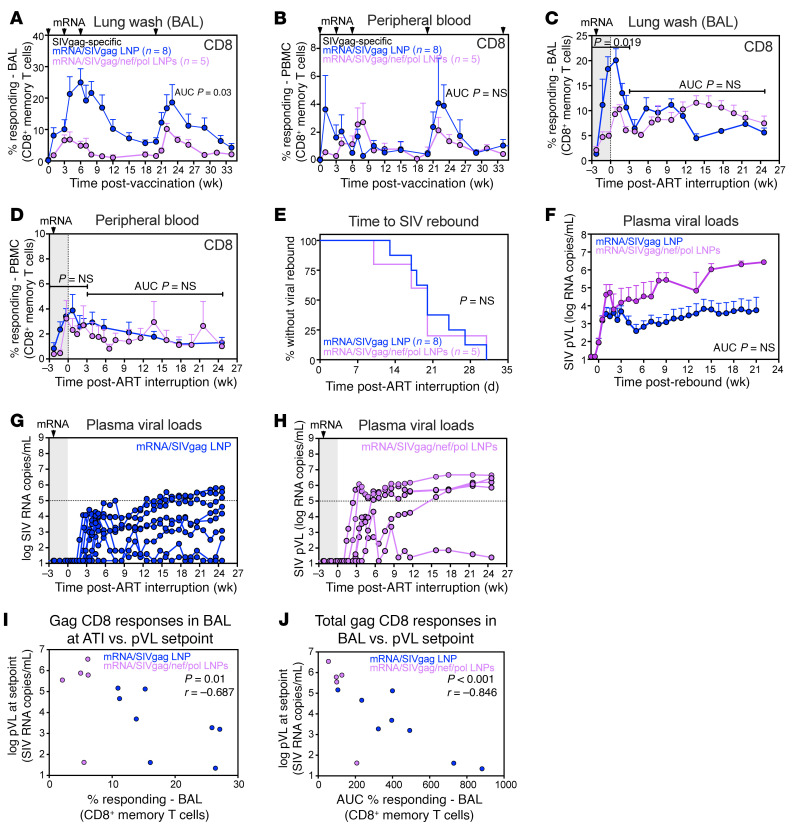
The addition of Nef and Pol vaccines reduced the magnitude of Gag-specific CD8^+^ T cell responses induced by the mRNA/SIVgag vaccine. (**A** and **B**) Comparison of SIV Gag–specific CD8^+^ T cell responses in BAL (**A**) and blood (**B**) of RMs in the mRNA/SIVgag group (*n* = 8) versus the mRNA/SIVgag/nef/pol group (*n* = 5) during ART. (**C** and **D**) SIV Gag–specific CD8^+^ T cell responses in BAL (**C**) and blood (**D**) of RMs in the same groups during ATI. Data represent mean frequencies (+SEM). (**E**) Kaplan-Meier analysis of SIV rebound kinetics in the mRNA/SIVgag group versus the mRNA/SIVgag/nef/pol group. (**F**) Mean (+SEM) PVL profiles of RMs in both groups following ATI. (**G** and **H**) Individual PVL trajectories of RM in each vaccine group after ATI. (**I**) Scatterplots of Gag-specific CD8^+^ T cell responses in BAL at the time of ATI versus PVL 24 weeks after ATI. (**J**) Scatterplots of total Gag-specific CD8^+^ T cell responses in BAL (measured from 0 to 34 weeks after the first vaccination) versus PVL 24 weeks after ATI. Statistical comparisons between vaccine groups were performed using Wilcoxon’s rank-sum test; *P* values ≤ 0.05 are indicated. Spearman’s rank correlation coefficients (*r*) with unadjusted *P* values are shown for scatterplots to assess associations between immune responses and viral loads.

Given that the mRNA/SIVgag/nef/pol vaccination did not enhance the effects observed with mRNA/SIVgag alone, we investigated whether this lack of enhancement was related to differences in the magnitude of Gag-specific CD8^+^ T cell responses following vaccination. To explore this, we compared the frequencies of Gag-specific CD8^+^ T cells in the blood and BAL of RMs that received either mRNA/SIVgag alone or mRNA/SIVgag in combination with mRNA/SIVnef and mRNA/SIVpol. As shown in Figure 7, A and B, and Supplemental Figure 8, Gag-specific CD8^+^ T cell responses were significantly higher in the BAL (*P* = 0.03) of mRNA/SIVgag-vaccinated RMs compared with those vaccinated with mRNA/SIVgag/nef/pol. Furthermore, after the final boost vaccination, the Gag-specific CD8^+^ T cell frequencies in the BAL of mRNA/SIVgag-vaccinated RMs were significantly higher (*P* = 0.019), peaking at a mean (+SEM) of 20.1% (+2.3%), compared with a mean (+SEM) of 10.3% (+0.9%) in the mRNA/SIVgag/nef/pol-vaccinated group (Figure 7C). Notably, although Nef- and Pol-specific CD8^+^ T cell responses in BAL were higher in the mRNA/SIVgag/nef/pol-vaccinated group during ART and after the final boost, the total CD8^+^ T cell response, calculated as the sum of Gag, Nef, and Pol responses, remained substantially higher in mRNA/SIVgag-vaccinated group (Supplemental Figure 8). However, no differences were observed in the frequencies of Gag-specific CD8^+^ T cells in the blood between the 2 vaccine groups (Figure 7D). Similarly, Gag-specific CD4^+^ T cell responses during ATI were not statistically different between groups (Supplemental Figure 9). Binding antibody responses to recombinant SIV Gag (p27) also showed no substantial differences between groups, either following vaccination or during ATI (Supplemental Figure 10), suggesting that B cell responses were not markedly perturbed.

Collectively, these results suggest that the inclusion of additional antigens in the vaccine protocol reduced the Gag-specific CD8^+^ T cell responses in the BAL (representing an immune effector site) compared with mRNA/SIVgag vaccination by itself. To determine whether this apparent antigenic interference in vaccine responses was specific to Gag, we conducted a pilot study in which a separate cohort of SIV-infected RMs on ART received either mRNA/SIVnef or mRNA/SIVpol (Supplemental Table 3). CD8^+^ T cell responses to Nef and Pol were then compared with those in animals vaccinated with mRNA/SIVgag/nef/pol. Overall, Nef- and Pol-specific CD8^+^ T cell responses in BAL were higher in RMs that received mRNA/SIVnef or mRNA/SIVpol alone than in those that received mRNA/SIVgag/nef/pol (Supplemental Figure 11). While the differences in the magnitude of Gag-specific CD8^+^ T cell responses between both vaccine groups did not have a significant impact on the time to measurable rebound viremia (Figure 7E), post-ART PVLs were higher in the mRNA/SIVgag/nef/pol group compared with the mRNA/SIVgag-only group (Figure 7F). Indeed, by 24 weeks after ART discontinuation, 80% of RMs (4 of 5) in the mRNA/SIVgag/nef/pol group exhibited PVLs > 5 logs compared with 37.5% of RMs (3 of 8) in the mRNA/SIVgag-alone group (Figure 7, G and H). When we explored correlates of post-ART viral control, a significant correlation was found between the magnitude of Gag-specific CD8^+^ T cells in the BAL, both the total frequencies measured by AUC between 0 and 34 weeks after the first vaccination (*P* < 0.001) and frequencies at the time of ATI (*P* = 0.01), and post-ART PVL set points 24 weeks after ATI (Figure 7, I and J). In contrast, there was no correlation between Gag-specific CD4^+^ T cells and post-ART viral load set points (Supplemental Figure 12). These findings suggest that the magnitude of vaccine-induced, Gag-specific CD8^+^ T cell responses in a mucosal tissue compartment such as the BAL influenced levels of SIVmac239 RNA in plasma 6 months following ATI.

## Discussion

CD8^+^ T cells play a critical role in combating intracellular pathogens such as HIV, with the ability in some individuals to mediate stringent control of viral replication. Consequently, strategies aimed at enhancing CD8^+^ T cell responses in PWH may offer an effective approach for achieving durable control of HIV replication following ART discontinuation. However, except for the role of responses to functionally constrained epitopes presented by protective MHC-Ia alleles, the immunologic characteristics of CD8^+^ T cell responses that can mediate sustained post-ART viral control are not well defined. In this study, we explored whether a therapeutic vaccination strategy designed to stimulate high frequencies of SIV-specific CD8^+^ T cell responses during ART followed by a boost just prior to ATI could substantially affect post-ART viral dynamics. The underlying hypothesis was that if timed correctly, boosting CD8^+^ T cell responses just before ATI would enhance immune interception of the virus at the initial stages of post-ATI viral rebound, prior to exponential viral expansion, and thereby promote long-lasting, immune-mediated control. To test this, we used an mRNA-based vaccine expressing either full-length SIV Gag or a combination of SIV Gag, Nef, and Pol. Of note, a key distinction between our study and other therapeutic vaccine approaches is that we administered the final boost immediately prior to ATI, ensuring that peak CD8^+^ T cell responses coincided with the expected timing of viral rebound. Indeed, boosting SIV-specific CD8^+^ T cell responses during ATI had a substantial effect on post-ART viral dynamics. Most notably, the statistically significant delay in the time to detectable viremia in the actively vaccinated animals underscores the observation that CD8^+^ T cells can be effective at combating foci of reactivating SIV infections.

These findings are important for several reasons. First, they provide clear evidence that activated SIV-specific CD8^+^ T cells, present in sufficient numbers at the time of ATI, can impede viral outgrowth from tissue sites that contribute to viral rebound, initially limiting viral spread. Second, our study confirms that naturally occurring CD8^+^ T cells are often not present in sufficient magnitude and/or at the necessary level of activation/differentiation/functional activity during ATI to control infection to the level needed to prevent pathogenesis, corroborating previous observations in SIVmac239-infected RMs that were depleted of CD8^+^ T cells during ATI (10). While the precise mechanisms underlying posttreatment control in a rare subset of PWH remain unclear (30), in most cases, rebound viremia is not effectively controlled until CD8^+^ T cell responses have undergone antigen-driven expansion in response to viral replication and spread (10), suggesting that this posttreatment control is due to the unique presence of more effective responses (epitope targeting, proliferative, and effector capabilities), rather than faster response dynamics. Therefore, our studies provide proof of concept for an approach whereby therapeutic HIV vaccines are used to induce the expansion of virus-specific CD8^+^ T cells earlier, prior to rebound, with the aim of restraining viral outgrowth, including in individuals without uniquely effective responses.

Third, our data demonstrate the importance of Gag-specific CD8^+^ T cell responses in promoting virologic control (23). Specifically, we found that Gag-specific CD8^+^ T cells elicited by mRNA/SIVgag were sufficient to induce a measurable delay in the time to viral rebound. As described earlier, the mRNA/SIVgag vaccine was designed to express full-length Gag, which is significant because full-length immunogens have been shown to induce CD8^+^ T cell responses of comparable breadth and magnitude against conserved epitopes, when compared with vaccines designed with conserved-region-only immunogens (31). However, whether the CD8^+^ T cell response should focus solely on Gag or continue to incorporate other viral proteins such as Nef, Pol, or Vif, and if so, in what manner to avoid antigenic interference, remains to be determined and warrants further investigation. Indeed, while the mRNA/SIVgag vaccine was highly immunogenic in SIV-infected RMs on ART, inducing robust Gag-specific CD8^+^ T cell responses, coimmunization with vaccines expressing Nef and Pol reduced the magnitude of Gag-specific CD8^+^ T cells observed in mucosal tissue sites, as represented by BAL. Although the exact mechanisms driving this reduction remain unclear, antigen competition may be a contributing factor. Notably, a randomized phase I clinical trial evaluating coimmunization of Ad5 vaccine vectors expressing Gag/Pol and Env found that Ad5 Env vectors reduced both the magnitude and breadth of Gag/Pol-specific CD4^+^ T cell responses, as well as the functionality of Gag-specific CD8^+^ T cell responses compared with immunizing with Ad5 Gag/Pol alone (32). This suggests that antigenic interference may not be unique to the mRNA-based vaccine platform used in this study.

While boosting frequencies of SIV-specific CD8^+^ T cells was effective in delaying viral rebound and restricting early viral replication, it did not lead to sustained post-ART viral control. In fact, viral rebound occurred in most RMs once CD8^+^ T cell response frequencies declined, and their persistence was not sustained by increased viral replication following rebound. This decline in Gag-specific CD8^+^ T cells could reflect a ceiling effect, where maximum expansion was reached due to limited immune compartment capacity (33, 34) during the interval between boost and rebound. As such, it is likely that our pre-ATI boost at 2 weeks before ATI was too early and that a slightly later boost, with later peak responses, might better cover the critical time period after rebound needed for durable control. Alternatively, rapid consumption of common γ chain cytokines may have constrained further expansion, suggesting that exogenous administration of these cytokines or other T cell regulators could potentially prolong the persistence of the SIV-specific CD8^+^ T cells (35–37) (see below). A caveat of this strategy is that these cytokines could also stimulate CD4^+^ T cells, which might inadvertently promote virus replication by target cell expansion. However, consistent with the approach of promoting T cell expansion, enhanced CD8^+^ T cell proliferation has been linked with post-ART viral control in previous studies (38, 39). Indeed, in a recent HIV cure clinical trial, participants with lower PVL set points after receiving a DNA vaccine expressing HIV Gag conserved elements, followed by an MVA vaccine, the TLR9 agonist lefitolimod, and 2 broadly neutralizing antibodies, showed enhanced CD8^+^ T cell proliferation in response to early post-ART viremia (30, 40). In addition, early ART was found to promote posttreatment control of SIV replication in RMs by inducing the development of CD8^+^ memory T cells with enhanced proliferative capacity (41). Mathematical modeling also supports this concept by showing that posttreatment controllers and noncontrollers can be distinguished by the rate of effector cell expansion (42).

It remains uncertain whether highly proliferative CD8^+^ T cells can be induced by vaccination in most PWH; however, inhibitors of glycogen synthase kinase-3 (GSK-3) have been shown to improve the functional capacity of HIV-specific CD8^+^ T cells, including upregulating the transcription factor, T cell factor 1 (TCF-1) (43). TCF-1 is a crucial regulator of memory CD8^+^ T cell proliferation and is highly expressed on stem-like memory T cells, which have a greater capacity for expansion (44, 45). HIV-specific CD8^+^ memory T cells from elite controllers exhibit higher levels of TCF-1 compared with those from PWH on ART or viremic noncontrollers (46). Thus, utilizing GSK-3 inhibitors to induce TCF-1 during vaccination may promote an expanded pool of virus-specific, stem-like memory T cells with high expansion potential. Combining IFN-I signaling blockade with an mRNA-based LNP vaccine has also been found to increase stem-like TCF-1^+^CD8^+^ T cell differentiation and improve vaccine efficacy (47), and it may offer an alternative approach to generating stem-like memory CD8^+^ T cells in the context of a therapeutic HIV/SIV vaccine. Interestingly, the live attenuated yellow fever vaccine (YF-17D) has been shown to induce stem-like memory T cells that persist for decades after vaccination (48), which may contribute to the durable protective efficacy of this vaccine.

Finally, we observed that the frequencies of Gag-specific CD8^+^ T cells in the BAL significantly correlated with post-ART PVL set points. The BAL, which samples cells in the pulmonary airspace, reflects a population of effector T cells that are continually replenished by the influx of proliferating memory T cells from other sites (49). As such, BAL responses serve as an indirect measure of overall effector T cell production (50). This suggests that vaccination strategies aimed at enhancing CD8^+^ effector T cell production, reflected here by BAL responses, may be effective in promoting durable post-ART viral control. Such strategies might include an additional booster vaccination after ATI or adjusting the timing of the final vaccination to ensure that responses peak closer to viral rebound. It is important to note that excessive antigen stimulation could lead to T cell exhaustion, limiting the ability of CD8^+^ T cells to effectively control viral rebound. This risk, however, could be mitigated through the use of immune checkpoint inhibitors, such as anti–PD-1 mAbs (51, 52). An alternative approach could involve administering HIV/SIV broadly neutralizing mAbs during ATI to modulate post-ART viral outgrowth, in combination with a therapeutic vaccine to stimulate CD8^+^ T cell expansion. In this context, vaccine-induced CD8^+^ T cell responses may be sufficient to control rebounding viremia. Indeed, a shift in the kinetics between viral outgrowth and the CD8^+^ T cell responses may account for the prolonged suppression of viremia often observed with broadly neutralizing mAb administration during ATI (53, 54).

We acknowledge that our study has several limitations (1). ART was initiated early after SIV infection (day 9). While this approach allows for systemic establishment of a saturated, rebound-competent viral reservoir (55), it differs from the typical clinical scenario in which most PWH initiate treatment during chronic HIV infection. While immediate ART initiation is now the recommended treatment guideline (56), the direct translatability of these findings to chronically treated PWH remains uncertain (2). Alongside the waning of SIV-specific CD8^+^ T cells, we also observed a decline in SIV-specific CD4^+^ T cells after viral rebound. Whether this reflects preferential infection of activated CD4^+^ target cells or other mechanisms that may have similarly impacted CD8^+^ T cell persistence (e.g., restricted immunological space, limited homeostatic cytokines) is unclear. It is also possible that the loss of CD8^+^ T cell responses was driven, at least in part, by reductions in CD4^+^ T helper cell support (3). Our studies did not specifically exclude RMs with protective MHC-I alleles. Thus, the potential contribution of protective MHC-I alleles to the observed antigenic competition or vaccine efficacy remains uncertain (4). It also remains to be determined whether the vaccination regimen can be further optimized to require fewer doses.

In summary, our studies highlight the effectiveness of CD8^+^ T cells in mediating an early immune intercept of reactivating SIV reservoirs during ATI. Although the mRNA/SIVgag vaccine restricted early viral spread, it was not sufficient to completely prevent rebound or induce durable posttreatment control. Nevertheless, these findings emphasize the potential of T cell–targeting vaccination strategies to mount a more timely and potent response to viral rebound, with the goal of facilitating durable virologic remission.

## Methods

### Sex as a biological variable.

This study included both male and female RMs. In the study comparing mRNA/SIVgag and mRNA/control vaccine, female RMs constituted 50% of animals in each group. In the study with the mRNA/SIVgag/nef/pol combination, only 1 of 10 animals was female. In the study with mRNA/SIVnef and mRNA/SIVpol alone, only male animals were used. Sex was not considered as a biological variable due to the small sample size.

### Animals, infections, and treatment.

Thirty-two male and female Indian rhesus macaques (*Macaca mulatta*) were used in this study. All RMs were specific pathogen free as defined by being free of *Simplexvirus macacinealpha1*, simian betaretrovirus, simian T-lymphotropic virus type 1, simian immunodeficiency virus, and *Mycobacterium tuberculosis* at study initiation. RMs were sequenced for common MHC-1 haplotypes (*Mamu* A*01, *Mamu* A*02, *Mamu* B*08, and *Mamu* B*17) using sequence-specific priming PCR as previously described (4). A cohort of 16 RMs was i.v. inoculated with 5,000 IU of the barcoded SIVmac239M before starting ART. ART consisted of daily s.c. injections of 5.1 mg/kg/d tenofovir disoproxil, 40 mg/kg/d emtricitabine, and 2.5 mg/kg/d dolutegravir in a solution containing 15% (v/v) kleptose at pH 4.2, as previously described (57), at 9 dpi through 758 dpi. RMs then received i.m. injections of mRNA/SIVgag (*n* = 8) or mRNA/control vaccine (*n* = 8) at 100 μg/dose at 59, 62, 65, 79, and 106 wpi, prior to ART discontinuation approximately 108 wpi. An additional 10 RMs were i.v. inoculated with 5,000 IU of SIVmac239M before starting ART at 9 dpi through 492 dpi. RMs then received i.m. injections of mRNA/SIVgag, mRNA/SIVnef, and mRNA/SIVpol vaccines (*n* = 5) at 100 μg/vaccine (300 μg in total) at 34, 37, 40, 54, and 68 wpi or no treatment (*n* = 5), prior to ART discontinuation approximately 70 wpi. An additional 6 RMs were i.v. inoculated with 5,000 IU of SIVmac239M before starting ART at 9 dpi through 933 dpi. RMs were divided into 2 groups that received i.m. injections of mRNA/SIVnef (*n* = 3) or mRNA/ SIVpol vaccines (*n* = 3) at 100 μg/dose at 59, 62, 65, and 79 wpi.

### mRNA vaccines.

The mRNA/SIVgag, mRNA/SIVnef, and mRNA/SIVpol vaccines are based on the RNActive platform (CureVac SE) and include no chemically modified nucleosides. The selection of this vaccine platform was based on previous studies demonstrating its ability to induce robust CD8^+^ T cell responses (58–60). The mRNAs encode SIVmac239 full-length Gag, Nef, and Pol protein (GenBank AY587015.1, AAW32418.1, and AAW32413.1, respectively). The mRNA control vaccine encoded the rabies virus glycoprotein (RABV-G), as previously described (24). mRNA vaccine designs have previously been described and contain a cleanCap followed by the 5′ UTR from the human hydroxysteroid 17-beta dehydrogenase 4 gene (*HSD17B4*) and a 3′ UTR from the human proteasome 20S subunit beta 3 gene (*PSMB3*) followed by a histone stem-loop and a poly(A)_100_ stretch (61, 62). The mRNA constructs were encapsulated in LNPs with LNP technology from Acuitas Therapeutics.

### Viruses.

The SIVmac239M challenge stock used in this experiment was produced by transfection of HEK239T cells, and the stock infectivity titer was determined using TZM-bl cells as previously described (27).

### SIV viral quantification assays.

Plasma SIV RNA levels were determined using a gag-targeted quantitative real-time/digital RT-PCR format assay, essentially as previously described providing an assay threshold of 15 SIV RNA copies/mL (63). Quantitative assessment of SIV DNA and RNA in cells and tissues was performed using gag-targeted, nested, quantitative, hybrid, real-time/digital RT-PCR and PCR assays, as previously described (2, 10, 64).

### Barcode sequencing.

Barcode sequencing was performed as previously described (27, 65). Briefly, RNA was isolated from plasma using the QIAamp Viral RNA mini kit (Qiagen) per the manufacturer’s instructions. cDNA was then synthesized from the extracted RNA using SuperScript III reverse transcriptase (Invitrogen) and a reverse primer (Vpr.cDNA3: 5′-CAGGTTGGCCGATTCTGGAGTGGATGC-3′). RT-qPCR was used to quantify the cDNA using the primers VpxF1 5′-CTAGGGGAAGGACATGGGGCAGG-3′ at 6082–6101 and VprR1 5′-CCAGAACCTCCACTACCCATTCATC-3′ at 6220–6199 and a fluorescently labeled probe (ACCTCCAGAAAATGAAGGACCACAAAGGG). Known quantities of the viral template were then PCR amplified with the same VpxF1 and VprR1 primers but with MiSeq adaptors directly synthesized onto the primers. Reactions were prepared using High Fidelity Platinum Taq (Thermo Fisher Scientific) per the manufacturer’s instructions, using primers VpxF1 and VprR1 with the following conditions: 94°C for 2 minutes followed by 40 cycles of 94°C, 15 seconds; 60°C, 90 seconds; 68°C, 30 seconds with final extension of 68°C for 5 minutes. Following PCR cleanup, amplicons were pooled and sequenced directly on the MiSeq instrument (Illumina).

### Flow cytometry.

Antigen-specific CD4^+^ and CD8^+^ T cell responses were measured in PBMCs, BAL, MLNs, PLNs, BM, spleen, liver, small intestine, and rectum by flow cytometric ICS for IFN-γ, TNF-α, and CD69 as previously described (2, 63). Briefly, mixes of sequential (11 amino acids overlapping) 15-mer peptides (AnaSpec) spanning the SIVmac239 Gag, Nef, and Pol open reading frames were used as antigens in conjunction with anti-CD28 (CD28.2, purified 500 ng/test; eBioscience, catalog 7014-0289-M050) and anti-CD49d stimulatory mAb (9F10, purified 500 ng/test; eBioscience, catalog 7014-0499-M050). Mononuclear cells were incubated at 37°C with peptide mixes and antibodies for 1 hour, followed by an additional 8 hours of incubation in the presence of Brefeldin A (5 μg/mL; Sigma-Aldrich). Stimulation in the absence of peptides served as background control. After incubation, stimulated cells were stored at 4°C until staining with combinations of fluorochrome-conjugated mAbs, including anti-CD3 (SP34-2, Pacific Blue, BD Biosciences, catalog 624034), anti-CD4 (L200, BV510, BD Biosciences, catalog 624144), anti-CD8a (SK1, PerCP-Cy5.5, BD Biosciences, catalog CUST05524), anti–IFN-γ (B27, APC, BioLegend, catalog 96018), anti-CD69 (FN50, PerCP-Cy5.5, BioLegend, catalog 93437), anti–TNF-α (MAB11, PE, BioLegend, catalog 96019), and anti-Ki67 (B56, FITC, BD Biosciences, catalog 624046).

SIV-specific CD8^+^ T cells were also measured by tetramer/hexamer staining. In brief, 100 μL whole blood or 0.5–2 × 10^6^ mononuclear cells from blood and tissues were stained with 100 ng of multimer (tetramer or dextramers). *Mamu* A*01^+^ tetramers consisted of Gag CM9 (CTPYDINQM) and Tat SL8 (STPESANL) biotinylated monomers conjugated with streptavidin BV421 and BV605, respectively. *Mamu* A*01^+^ Gag CM9 dextramers (JH02641 PE 15, Immudex) were purchased preconjugated with PE. Cells were stained with combinations of fluorochrome-conjugated surface antibodies, including anti-CD3 (SP34-2, BUV395, BD Biosciences, catalog 624310), anti-CD4 (L200, BUV786; BD Biosciences, catalog 624162), anti-CD8α (Sk-1, BUV737, BD Biosciences, catalog 624235), anti-CD28 (CD28.2, BV510, BioLegend, catalog 92582), anti-CD95 (DX2, PE, BioLegend, catalog 94203), anti-CCR7 (G043H7, BV711, BioLegend, catalog 92556), anti-CCR5 (3A9, APC, BD Biosciences, catalog 624346), anti-CD20 (2H7, Alexa Fluor 700, BioLegend, catalog 98488), and anti-Ki67 (B56, FITC, BD Biosciences, catalog 624046). MHC-peptide complexes were provided as biotinylated monomers by the NIH Tetramer Core Facility and conjugated with streptavidin-BV421 (BioLegend, catalog 92067) or streptavidin-BV605 (BioLegend, catalog 94109) in a 4:1 molar ratio and prepared at a final concentration of 50 ng/μL in PBS.

To determine the phenotype of T cells and monocytes, whole blood was stained as previously described (66). Cells were stained for 30 minutes, followed by washing and staining with combinations of fluorochrome-conjugated surface antibodies, including anti-CD3 (SP34, BUV395, BD Biosciences, catalog 624310), anti-CD4 (L200, BV510, BD Biosciences, catalog 624144), anti-CD8α (RPA-T8, BV711, BioLegend, catalog 900006277), anti-CD95 (DX2, BV605, BioLegend, catalog 305628), anti-CD28 (CD28.2, PEDazz594, BioLegend, catalog 93364), anti-CCR5 (3A9, APC, BD Biosciences, catalog 624346), anti-Ki67 (B56, FITC, BD Biosciences, catalog 624046), anti-CD14 (M5E2, BUV615, BD Biosciences, catalog 624297), anti-CD169 (7-239, PE, BioLegend, catalog 96746), anti-CD123 (7G3, BV786, BD Biosciences, catalog 624317), anti-CD16 (3G8, BUV496, BD Biosciences, catalog 624283), anti-CD11c (3.9, APC, BioLegend, catalog 950405), anti-HLA-DR (L243, PE Cy7, BioLegend, catalog 900002339), anti-CD20 (2H7, APC/Fire 750; BioLegend, catalog 93924), anti-CCR7 (G043H7, biotin, BioLegend, catalog 93747), and anti-streptavidin (BV421, BioLegend, catalog 92067). Monocyte activation was determined by expression of CD169 on CD14^+^HLA-DR^+^CD20^–^CD3^–^CD16^–^ cells (Supplemental Figure 1). List mode multiparameter data files were analyzed using the FlowJo software program (version 10.10.0; Tree Star).

### Luminex assay.

Frozen plasma samples, previously isolated and cryopreserved from whole blood, were used to quantify 27 cytokines and chemokines using the Cytokine 29-Plex Monkey Panel Kit (Thermo Fisher Scientific, catalog LPC0005M) according to the manufacturer’s instructions.

### Sample size and treatment assignment.

Sample size was determined by logistical and resource considerations. Treatment assignments (mRNA/SIVgag vs. mRNA/control and mRNA/SIVgag/nef/pol vs. no treatment) were balanced based on sex, age, and presence of protective MHC alleles. No blinding was possible due to the constraints of working with RMs.

Additional information may be found in Supplemental Methods.

### Artificial intelligence tools.

During the writing of this manuscript, OpenAI’s ChatGPT (version ChatGPT-4) was used to check grammar, spelling, and/or the clarity of phrases or sentences. Prompts used when working with the tool included “edit this sentence or phrase.” The artificial intelligence (AI) tool suggested alternatives that were reviewed and edited as needed. The authors take full responsibility for the content of the publication.

### Statistics.

Box-and-whisker plots show jittered points, a box from first to third quartiles (IQR), and a line at the median, with whiskers down to the minimum and up to the maximum value. We used 2-sided Wilcoxon’s rank-sum tests for all analyses comparing values across treatment groups. Point values were transformed to the log_10_ scale where indicated. For analyses involving multiple time points, we calculated the AUC or peak value for each RM and analyzed the resulting values in a fashion similar to that using single–time point data. The AUC was calculated either over the whole curve or specific ranges, as specified in the figure legends. To compare time-to-event data, we used the log-rank test. We used Spearman’s rank transformation when evaluating correlations. All tests were conducted with a 2-sided null hypotheses at significance level *P* ≤ 0.05. All analyses were performed in Python 3.12.7 with the package scipy 1.14.1. While we presented primarily unadjusted *P* values in accordance with our prespecified plan, and consistent with our usual practice, we value and encourage consideration of the impact of multiple testing. For post hoc multiplicity adjustment over any set of tests, we provide all unadjusted *P* values in the Supporting Data Values file. All plots were created using GraphPad Prism version 10.4.1.

### Study approval.

Animal use was approved by Oregon National Primate Research Center’s IACUC according to the NIH’s Guide for the Care and Use of Laboratory Animals.

### Data availability.

Data used to generate figures are provided in the Supporting Data Values file. All SIV sequencing data have been deposited in GenBank under accession numbers PX559744–PX559797. Any additional information required to reanalyze the data reported in the paper is available from the corresponding author upon request.

## Author contributions

AAO and LJP conceived and planned the study and wrote the paper. WRO and BDVM supervised RM experiments and analyzed immunological and virological data, assisted by OF, AM, DMD, WDG, PA, and HT. JDL quantified SIV data, assisted by WJB, RF, CH, KO, and RS. BFK, CMF, E Kose, and TTI performed SIVmac239M barcode sequencing and analysis as well as full genome sequencing. JVS and MKA managed the animal protocols, assisted by CSL, RB, and RM. JG, BP, and SR provided vaccines. PTE and E Kosmider conducted statistical analyses.

## Funding support

This work is the result of NIH funding, in whole or in part, and is subject to the NIH Public Access Policy. Through acceptance of this federal funding, the NIH has been given a right to make the work publicly available in PubMed Central.

- Gilead Sciences Cure grant (05040 to AAO).
- National Institute of Allergy and Infectious Diseases (R01 AI152609 to AAO and UM1 AI164560 to LJP).
- NIH Office of the Director (P51OD011092) for operation of the Oregon National Primate Research Center.
- National Cancer Institute funding, in whole or in part, under contract 75N91019D00024 (to JDL and BFK).

## Supplementary Material

Supplemental data

Supporting data values

## Version 1 01/29/2026

In-Press Preview

## Version 2 03/16/2026

Electronic publication
