# Supplementary material for: Boosting SIV-specific CD8+ T cell responses prior to ART interruption extends time to SIVmac239 rebound
Source: J Clin Invest. 2026 Jan 29;136(6):e198294. doi: 10.1172/JCI198294 (PMC12987612; doi:10.1172/JCI198294)
Supplement: Supplemental data [file jci-136-198294-s143.pdf]

Supplemental Materials

Boosting SIV-specific CD8$^+$ T cell responses prior to ART interruption extends time to SIVmac239 rebound

Were R. Omange, Benjamin D. Varco-Merth, Omo Fadeyi, Alejandra Marenco, Hiroshi Takata, Derick M. Duell, William Goodwin, Paula Armitage, Christine M. Fennessey, Emek Kose, Taina T Immonen, Ewelina Kosmider, William J. Bosche, Randy Fast, Chris Homick, Kelli Oswald, Rebecca Shoemaker, Rachele Bochart, Rhonda MacAllister, Caralyn Labriola, Jeremy V. Smedley, Michael K. Axthelm, Paul T. Edlefsen, Brandon F. Keele, Jeffrey D. Lifson, Janina Gergen, Benjamin Petsch, Susanne Rauch, Louis J. Picker and Afam A. Okoye.

Correspondence address to: Afam A. Okoye, Vaccine and Gene Therapy Institute, Oregon Health & Science University, West Campus, 505 NW 185th Ave., Beaverton, Oregon 97006, USA. Phone: 503.418.2752; Email: okoyea@ohsu.edu.

Supplemental Methods

Near full-length single genome sequencing. Near full-length sequencing was performed as described previously (Long et al, 2019 JV). Briefly, viral RNA was isolated from plasma using QIAamp Viral RNA kit (Qiagen). Extracted RNA was reverse transcribed into cDNA using SuperScript III (Qiagen) and the viral specific primer nFL-R1 (5'-CAC TAG CTT ACT TCT AAA ATG GCA GC). cDNA was then diluted to a single genome template prior to PCR with nFL-R1 and nFL-F1 (5'-GAT TGG CGC CYG AAC AGG GAC TTG) primers. Second round PCR was performed with nFL-R2 (5'-TAC TTC TAA AAT GGC AGC TTT ATT GAA) and nFL-F2 (5'- GTG AAG GCA GTA AGG GCG GCAGG) primers. Both PCR reactions were amplified with Platinum SuperFi DNA polymerase (ThermoFisher Scientific) and correct sized amplicons were directly sequencing using BigDye Terminator Sanger sequencing (ThermoFisher Scientific) with multiple virus-specific primers. Nucleotide sequences were deposited into GenBank (accession numbers PX559744-PX559797).

Measurement of SIV Gag-specific antibodies. Maxisorp 96-well Immunoplates (Thermo Fisher Scientific Nunc) were coated overnight at 4 °C with recombinant SIV Gag p27 (NIH BEI Resources) at 1 μg/mL in PBS. The following day, plates were washed five times with PBS containing 0.05% Tween-20 (MilliporeSigma; wash buffer) and blocked for 30 min at 37 °C with 5% normal goat serum in PBS. After an additional wash, plasma samples and an anti-SIV p27 monoclonal antibody (clone 55-2F12, NIH BEI Resources) standard were added in duplicate at serial dilutions and incubated for 2 h at room temperature (RT). Plates were then washed and incubated for 1 hour at RT with 1 μg/mL HRP-conjugated anti-monkey IgG (Fitzgerald) or anti-mouse IgG (Thermo Fisher Scientific Invitrogen) for the p27 standard curve. Following a final wash, 3,3',5,5'-Tetramethylbenzidine (TMB; MilliporeSigma) substrate was added until color developed, and the reaction was stopped with 1 M H_3PO_4. Absorbance was measured at 450 nm and 650 nm using a BioTek Synergy HTX Multimode Reader (Agilent). Concentrations were determined in

GraphPad Prism 9 using a four-parameter logistic fit of the standard curve. The mean of duplicate wells is reported as U/mL, with the p27 standard curve expressed in ng/mL.

Supplemental Figures and Tables

Animal ID	Sex	Treatment Group	Age at time of SIV infection (years)	Viral challenge strain (5000 IU)	Peak plasma viral loads (Log SIV RNA copies/ml)	ART START (days post-SIV infection)	Time on ART (days)	Known protective MHC-1 allele
A1	Male	mRNA/SIVgag	4.3	SIVmac239M	7.6	9	758	A*01
A2	Female	mRNA/SIVgag	3.7	SIVmac239M	6.7	9	758	B*17
A3	Female	mRNA/SIVgag	3.7	SIVmac239M	7.0	9	758	
A4	Female	mRNA/SIVgag	3.7	SIVmac239M	7.5	9	758	A*01, B*08
A5	Male	mRNA/SIVgag	5.0	SIVmac239M	6.9	9	758	B*17
A6	Male	mRNA/SIVgag	5.0	SIVmac239M	7.5	9	758	A*01, B*08
A7	Male	mRNA/SIVgag	4.5	SIVmac239M	7.3	9	758	B*17
A8	Female	mRNA/SIVgag	3.6	SIVmac239M	6.6	9	758	
B1	Male	mRNA/Control	4.8	SIVmac239M	7.0	9	785	
B2	Male	mRNA/Control	4.9	SIVmac239M	6.9	9	785	A*01, B*17
B3	Male	mRNA/Control	4.8	SIVmac239M	7.2	9	785	B*08
B4	Female	mRNA/Control	3.7	SIVmac239M	7.0	9	785	
B5	Female	mRNA/Control	4.7	SIVmac239M	7.3	9	785	B*08
B6	Female	mRNA/Control	4.2	SIVmac239M	7.8	9	785	
B7	Female	mRNA/Control	3.5	SIVmac239M	6.7	9	758	A*01
B8	Male	mRNA/Control	3.5	SIVmac239M	6.8	9	758	B*17

Supplemental Table 1. Characteristics of RM in the study comparing mRNA/SIVgag versus mRNA/Control vaccination. The table shows the sex, age at the time of SIV infection, challenge virus, peak plasma viral loads, duration of of ART relative to SIV infection and known protective MHC-1 alleles.

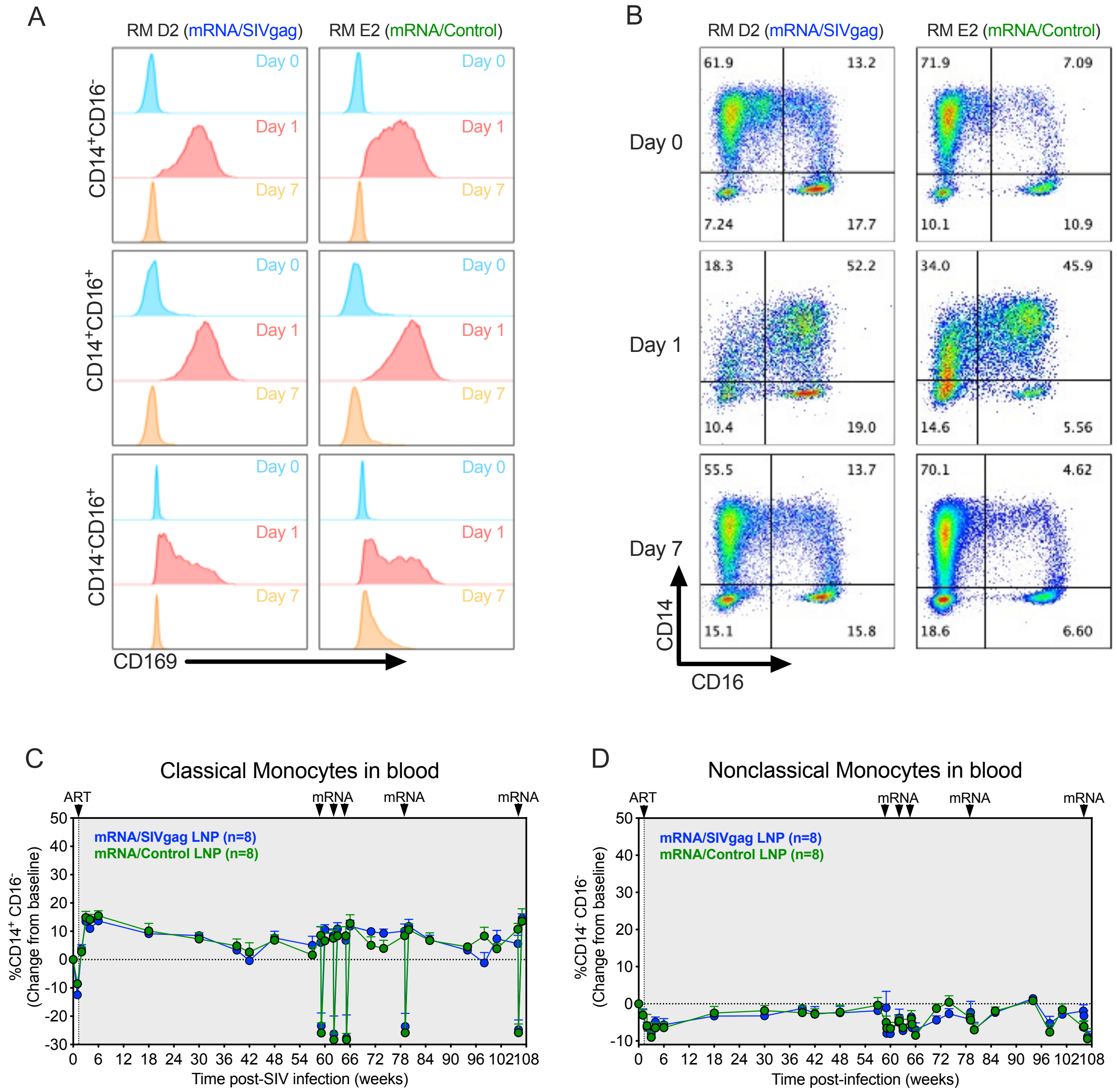

Supplemental Figure 1. Effects of mRNA vaccines on monocytes. (**A**) Showing CD169 expression on monocyte subsets at days 0, 1, and 7 post-vaccination in representative RMs from each vaccine group. (**B**) Representative flow cytometry plots showing monocyte subsets in the blood of an RM from each vaccine group at days 0, 1, and 7 post-vaccination. (**C**) Mean (+ SEM) frequencies of CD14^{+}CD16^{+} monocytes, and (**D**) CD14^{-}CD16^{-} monocytes, shown as a fraction of CD3^{-}, CD20^{-}, CD8^{-} and HLA-DR^{+} lymphocytes in the blood of RMs from the mRNA/SIVgag vaccine group (n = 8) and the mRNA/Control group (n = 8), prior to ART discontinuation.

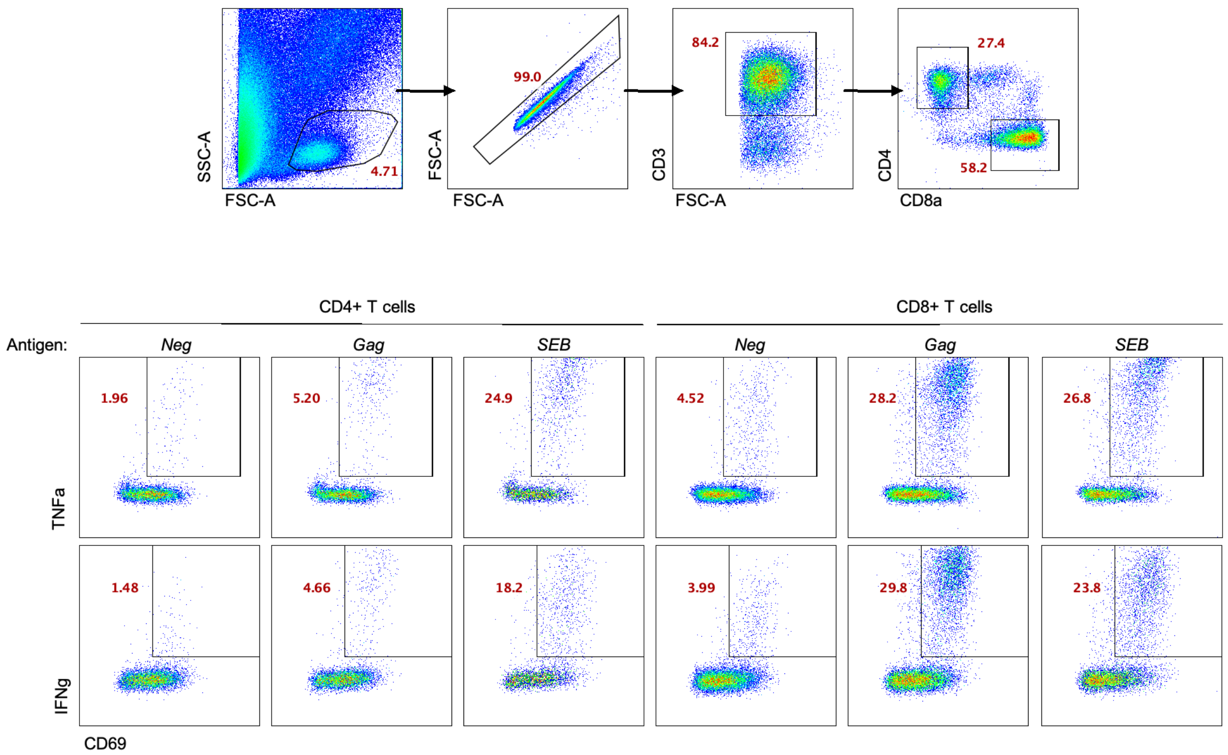

Supplemental Figure 2. Gating strategy for flow cytometry intracellular cytokine analysis. Cells were stimulated, stained and collected on a flow cytometer as described in the Methods. Data was analyzed using a hierarchical gating strategy to delineate $CD4^+$ and $CD8^+$ T cell populations followed by determination of the induced cytokine production within these populations. Shown here is the gating for $CD4^+$ and $CD8^+$ T cells in the BAL two weeks after the last mRNA/SIVgag vaccination. The total response values after antigen stimulation were determined using a Boolean gate, combining $CD69^+$ $TNF\alpha^+$ and/or $IFN\gamma^+$ events. The negative control represents T cells not exposed to antigen.

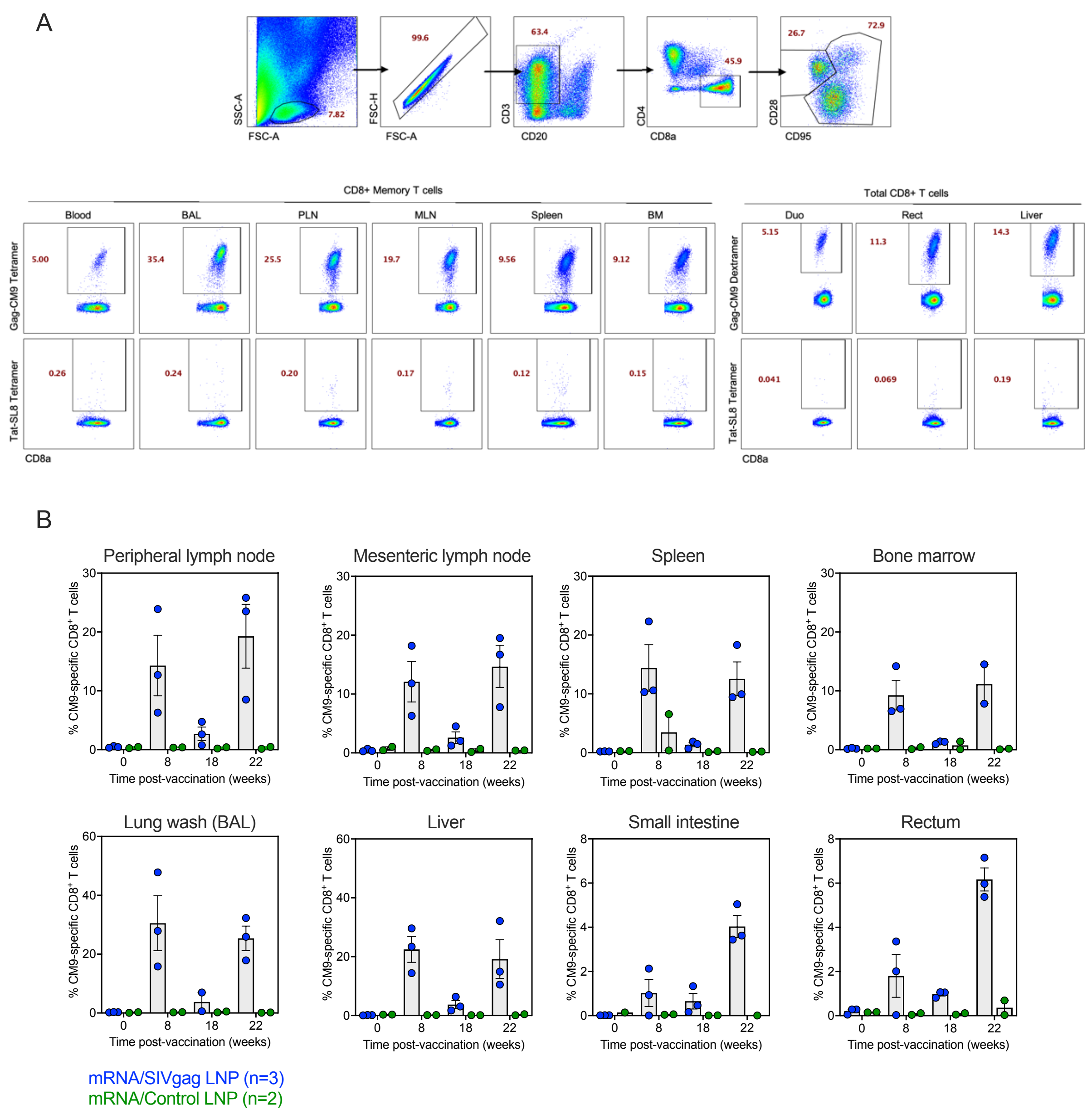

Supplemental Figure 3. Gag CM9 epitope-specific CD8^{+} T cell responses induced by the mRNA/SIVgag vaccine. (**A**) Gating strategy for SIV-specific CD8^{+} T cells. Cells were stained with MHC-I multimers and analyzed by flow cytometry as described in the Methods. A progressive gating strategy was used to identify CD3^{+} CD8^{+} CD95^{+} T cell populations, followed by quantification of SIV-specific cells within these subsets. Shown is the gating strategy for detecting Gag-CM9^{+} or Tat-SL8^{+} cells in the blood of a *Mamu*-A*01^{+} RM two weeks after the last mRNA/SIVgag vaccination. (**B**) Frequencies of CD8^{+} T cells specific for SIV Gag-CM9 measured in peripheral lymph nodes (PLN), mesenteric lymph nodes (MLN), spleen, bone marrow (BM), BAL, liver, small intestine (Duo) and rectum (Rect) from three mRNA/SIVgag-vaccinated and two mRNA/control *Mamu*-A*01^{+} animals.

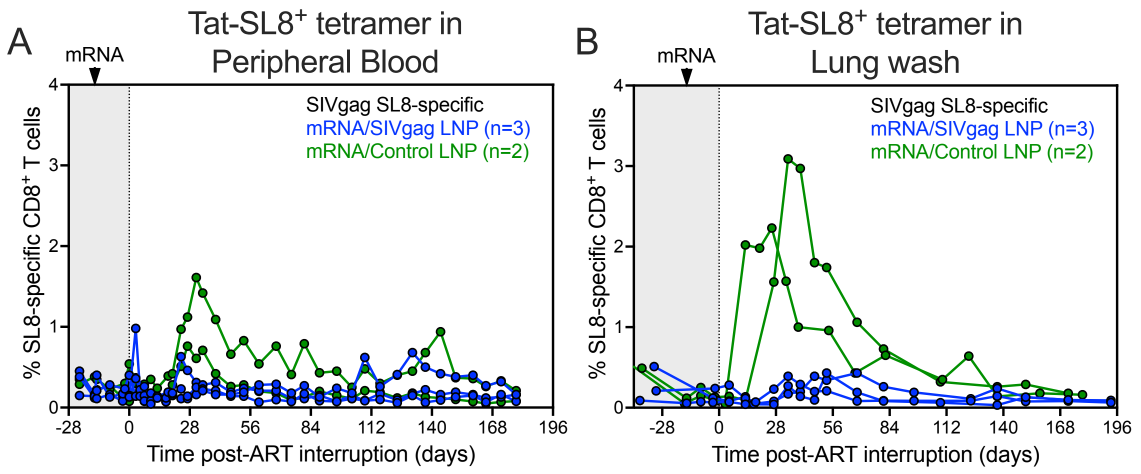

Supplemental Figure 4. Tat SL8 epitope-specific CD8⁺ T cell responses during ATI. Frequencies of CD8⁺ T cells specific for SIV Tat-SL8 measured in (**A**) peripheral blood and (**B**) BAL of three mRNA/SIVgag-vaccinated and two mRNA/control *Mamu*-A*01⁺ animals after ART interruption.

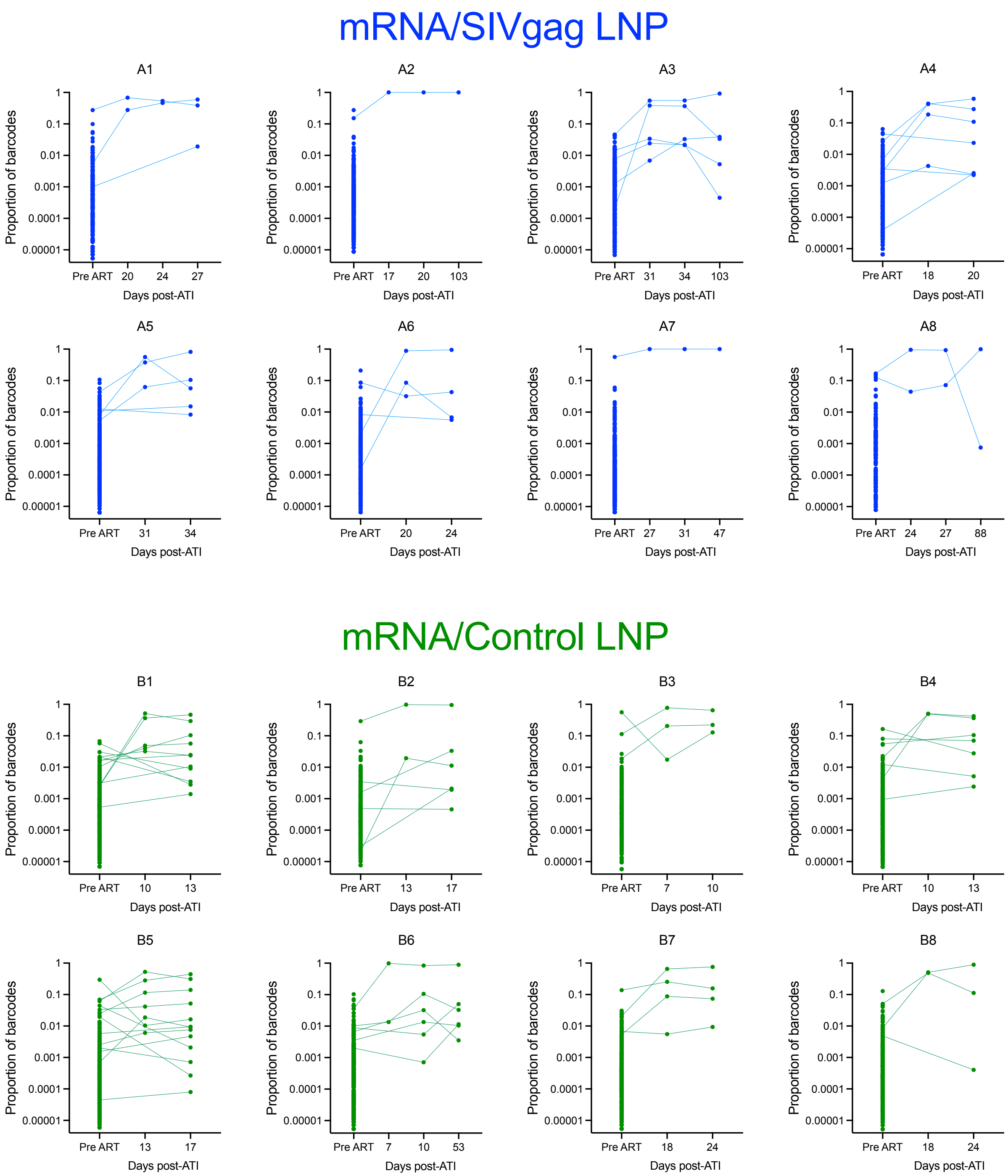

Supplemental Figure 5. Assessment of SIVmac239M barcodes post-rebound. Showing the relative barcode proportion at day of ART (9 days post-infection) and post-ART interruption for RM in the mRNA/SIVgag group (top panels) and mRNA/Control group (bottom panels).

A

A2 - pVL 45,000

Position	Gag 244	Vpr76	Rev40	Env90	Env751	Nef18	Nef16	Nef170	Nef195	Nef195
Reference sequence	SSVDEQIQW	MHFRGGCIH	TANQRRQRK	ETWYSADLV	GKERDGGEG	NPAEEREKL	NPAEEREKL	IRYPKTFGW	MHPAQTSQW	MHPAQTSQW
Mutation	SSVEEQIQW	MHFRGRCIH	TANQRRQRR	ETWYSTDLV	GKEGDGGEG	NPAEERKKL	NPAEKREKL	IRYPKIFGW	VHPAQTSQW	IHPAQTSQW
% of sequences	100	100	100	100	100	69	31	54	46	38

A3 - pVL 180,000

Position	Vif191	Vpx133	Vpx134	Rev40	Rev191	Env40	Env751	Env802	Nef167
Reference sequence	GDKQRGGKP	EINREAVNHL	EINREAVNHL	TANQRRQRK	IESIPDPPTNT	VPAWRNATI	REGKERDGG	ILQRLSATLQR	IRYPKTFGW
Mutation	GDKHRGGKP	EISREAVNHL	EINIEAVNHL	TANQRRQRR	IESIPDPPTNI	VPAWRNATV	REGKEGDGG	IFQRLSATLQR	IRFPKTFGW
% of sequences	40	55	35	100	45	30	100	45	100

A7 - pVL 4,100

Position	Vpr75	Tat25
Reference sequence	HFRGGCIHS	CISEADAST
Mutation	HFRDGCIHS	CISETDAST
% of sequences	100	100

A8 - pVL 130,000

Position	Gag244	Pol720	Vif190	Vpx32	Tat112	Tat118	Env747	Env753	Nef37
Reference sequence	SSVDEQIQW	QGIRQVLFLE	RGDKQRGGK	EINREAVNHL	EKAKKETVEK	EKAKKETVEK	REGKERDGGEG	REGKERDGGEG	LLGEVEDGYS
Mutation	SSVEEQIQW	RGIRQVLFLE	RGDEQRGGK	EMNREAVNHL	EEAKKETVEK	EKAKKETMEK	RGGKERDGGEG	REGKERDDGEG	LLGEVEGGYS
% of sequences	100	100	89	89	33	72	33	72	100

B

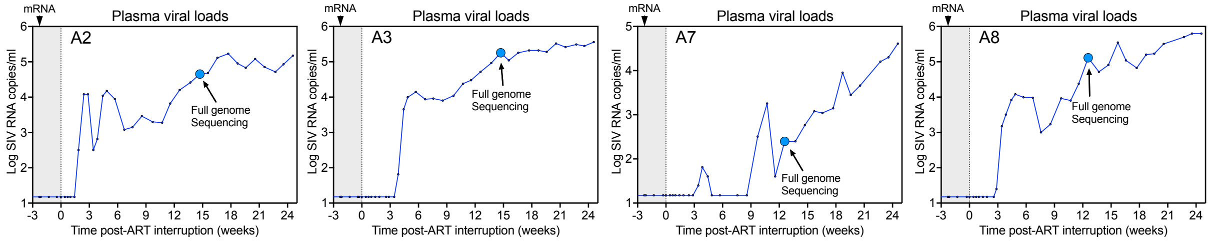

Supplemental Figure 6. Limited escape mutations in Gag in mRNA/SIVgag vaccinated RM. (**A**) Sequencing of SIV RNA from post-rebound plasma samples collected from four RM in the mRNA/SIVgag vaccine group was conducted to assess the frequency of escape mutations. Amino acid substitutions at specific sites are highlighted in red. Only mutations present at frequencies exceeding 30% within the plasma viral populations are shown. These nucleotide sequences were deposited into GenBank (accession numbers PX559744-PX559797). (**B**) Individual plasma viral load profiles of RM indicating the timing of sequencing relative to ART interruption.

Animal ID	Sex	Treatment Group	Age at time of SIV infection (years)	Viral challenge strain (5000 IU)	Peak plasma viral loads (Log SIV RNA copies/ml)	ART START (days post-SIV infection)	Time on ART (days)	Known protective MHC-1 allele
Y1	Male	mRNA/SIVgag, mRNA/SIVnef, mRNA/SIVpol	6.9	SIVmac239M	6.6	9	492	A*01+ B*17
Y2	Male	mRNA/SIVgag, mRNA/SIVnef, mRNA/SIVpol	5.9	SIVmac239M	6.6	9	492	
Y3	Male	mRNA/SIVgag, mRNA/SIVnef, mRNA/SIVpol	5.9	SIVmac239M	6.2	9	492	
Y4	Male	mRNA/SIVgag, mRNA/SIVnef, mRNA/SIVpol	6.0	SIVmac239M	7.0	9	492	
Y5	Male	mRNA/SIVgag, mRNA/SIVnef, mRNA/SIVpol	6.0	SIVmac239M	6.8	9	492	
X1	Male	Unvaccinated Controls	7.3	SIVmac239M	7.6	9	492	
X2	Male	Unvaccinated Controls	5.9	SIVmac239M	7.4	9	492	
X3	Male	Unvaccinated Controls	6.0	SIVmac239M	6.8	9	492	
X4	Male	Unvaccinated Controls	4.9	SIVmac239M	7.3	9	492	A*01
X5	Female	Unvaccinated Controls	7.1	SIVmac239M	6.9	9	492	

Supplemental Table 2. Characteristics of RM in the study comparing mRNA/SIVgag/nef/pol vaccination versus unvaccinated controls. The table shows the sex, age at the time of SIV infection, challenge virus, peak plasma viral loads, duration of of ART relative to SIV infection and known protective MHC-1 alleles.

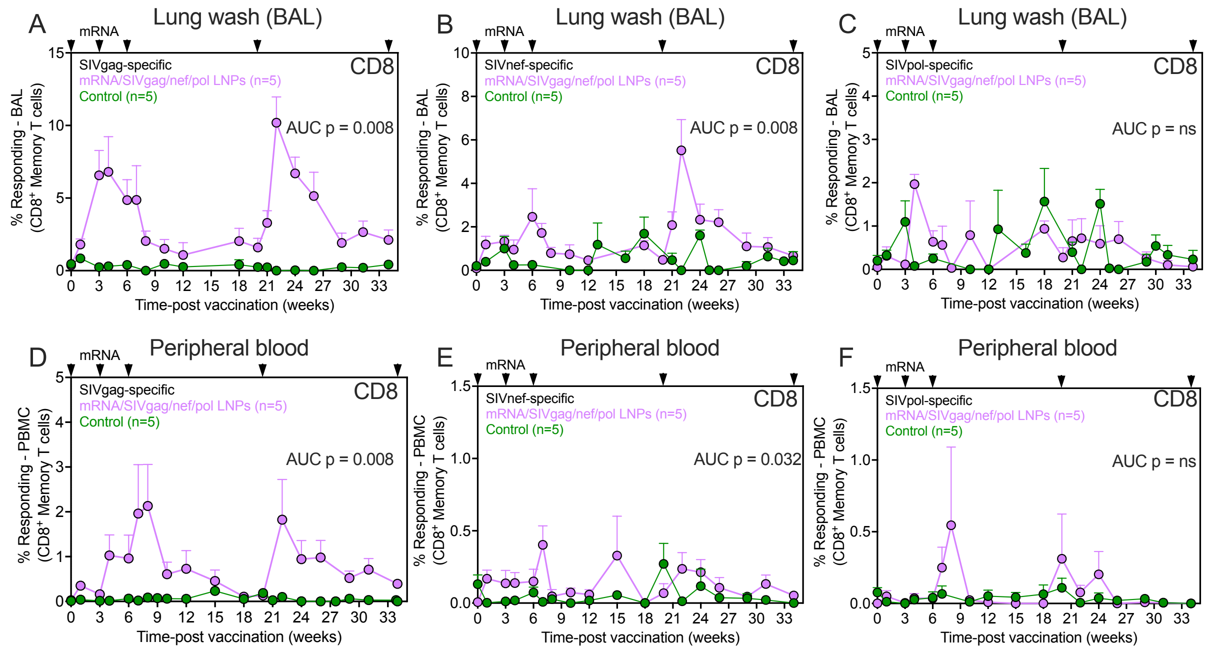

Supplemental Figure 7. Gag, Nef and Pol specific CD8$^+$ T cell responses induced by mRNA/SIVgag/nef/pol vaccination. (**A-C**) Comparison of SIV CD8$^+$ T cell responses to Gag (**A**), Nef (**B**), and Pol (**C**) in BAL of RM in the mRNA/SIVgag/nef/pol group versus controls during ART. (**D-F**) Comparison of SIV CD8$^+$ T cell responses to Gag (**D**), Nef (**E**), and Pol (**F**) in blood of RMs from the same groups during ART. Data represents mean frequencies (n = 5, + SEM). Statistical significance between groups was determined by Wilcoxon rank-sum (WRS) test; p-values ≤ 0.05 are indicated.

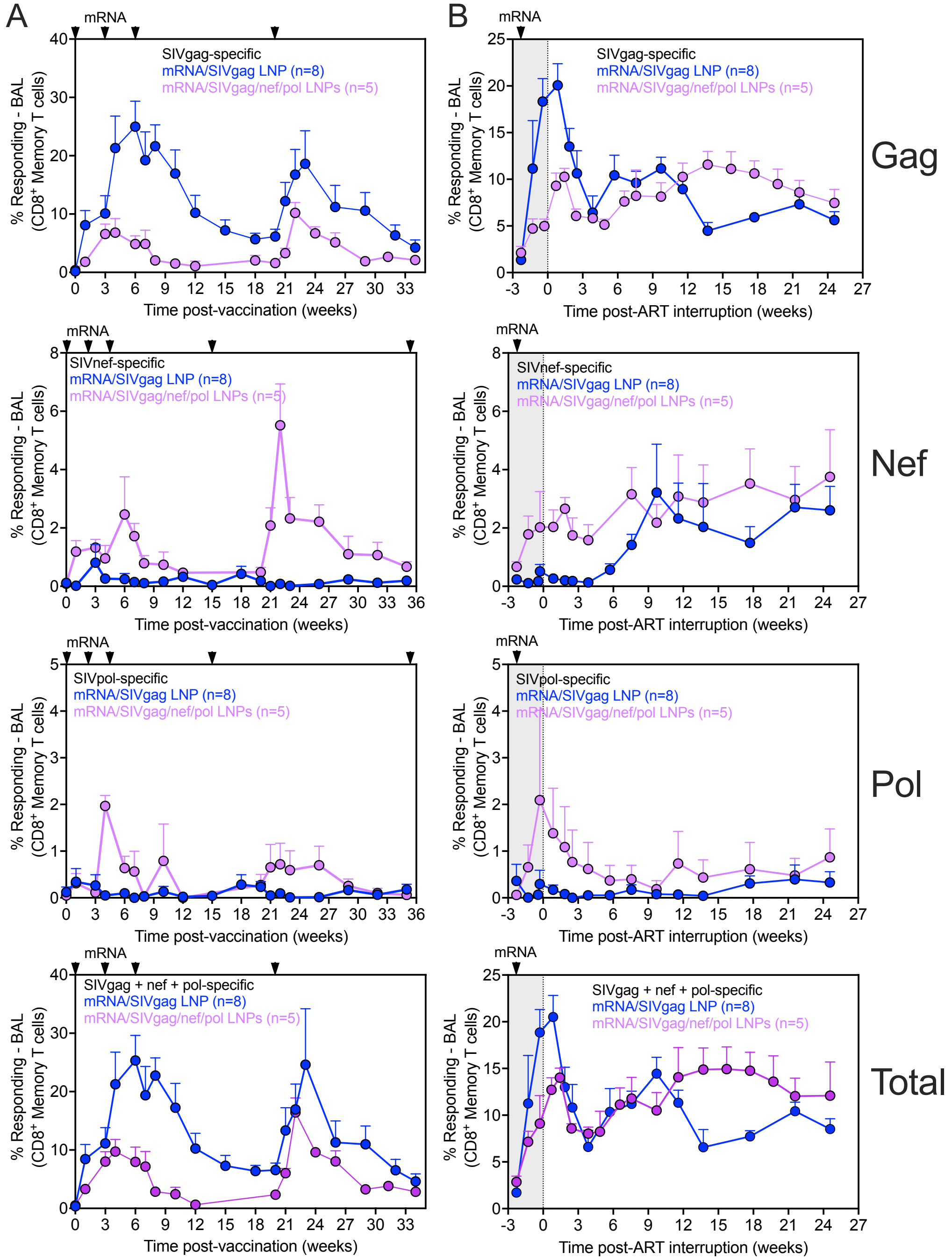

Supplemental Figure 8. Comparing Gag, Nef, Pol and total SIV-specific CD8$^+$ T cell responses in RM vaccinated with mRNA/SIVgag versus mRNA/SIVgag/nef/pol. (**A**) SIV-specific CD8$^+$ T cell responses to Gag, Nef, Pol and total (Gag + Nef + Pol) antigens in BAL of RM during ART in the mRNA/SIVgag group (n = 8) versus mRNA/SIVgag/nef/pol group (n = 5). (**B**) SIV-specific CD8$^+$ T cell responses to the same antigens in BAL of RM from the same groups during ART interruption. Statistical significance between groups was determined by Wilcoxon rank-sum (WRS) test; p-values ≤0.05 are indicated.

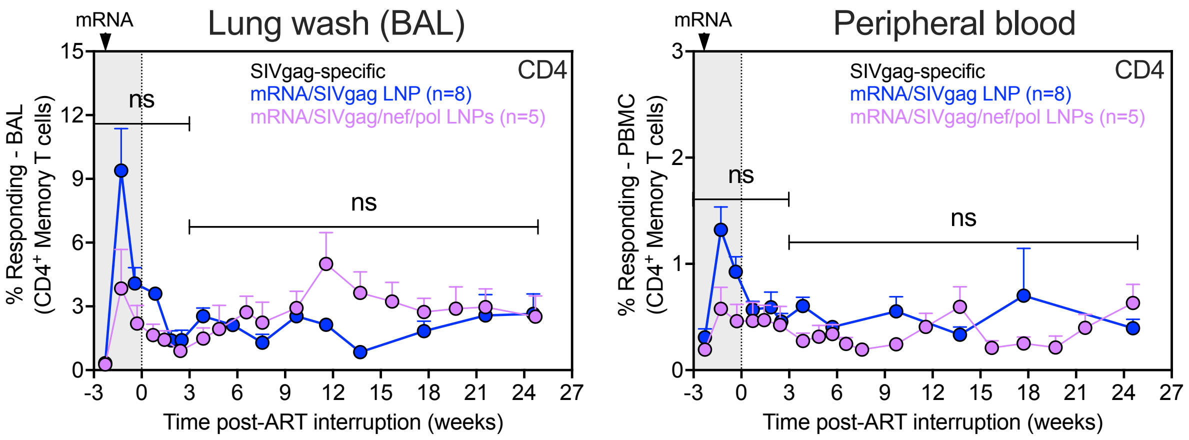

Supplemental Figure 9. Comparing Gag-specific CD4$^+$ T cell responses induced by mRNA/SIVgag versus mRNA/SIVgag/nef/pol vaccination. Comparison of SIV Gag-specific CD4$^+$ T cell responses in BAL (left panel) and blood (right panel) of RM in the mRNA/SIVgag group versus mRNA/SIVgag/nef/pol group during ART interruption. Data represents mean frequencies (+ SEM). Statistical significance between groups was determined by Wilcoxon rank-sum (WRS) test.

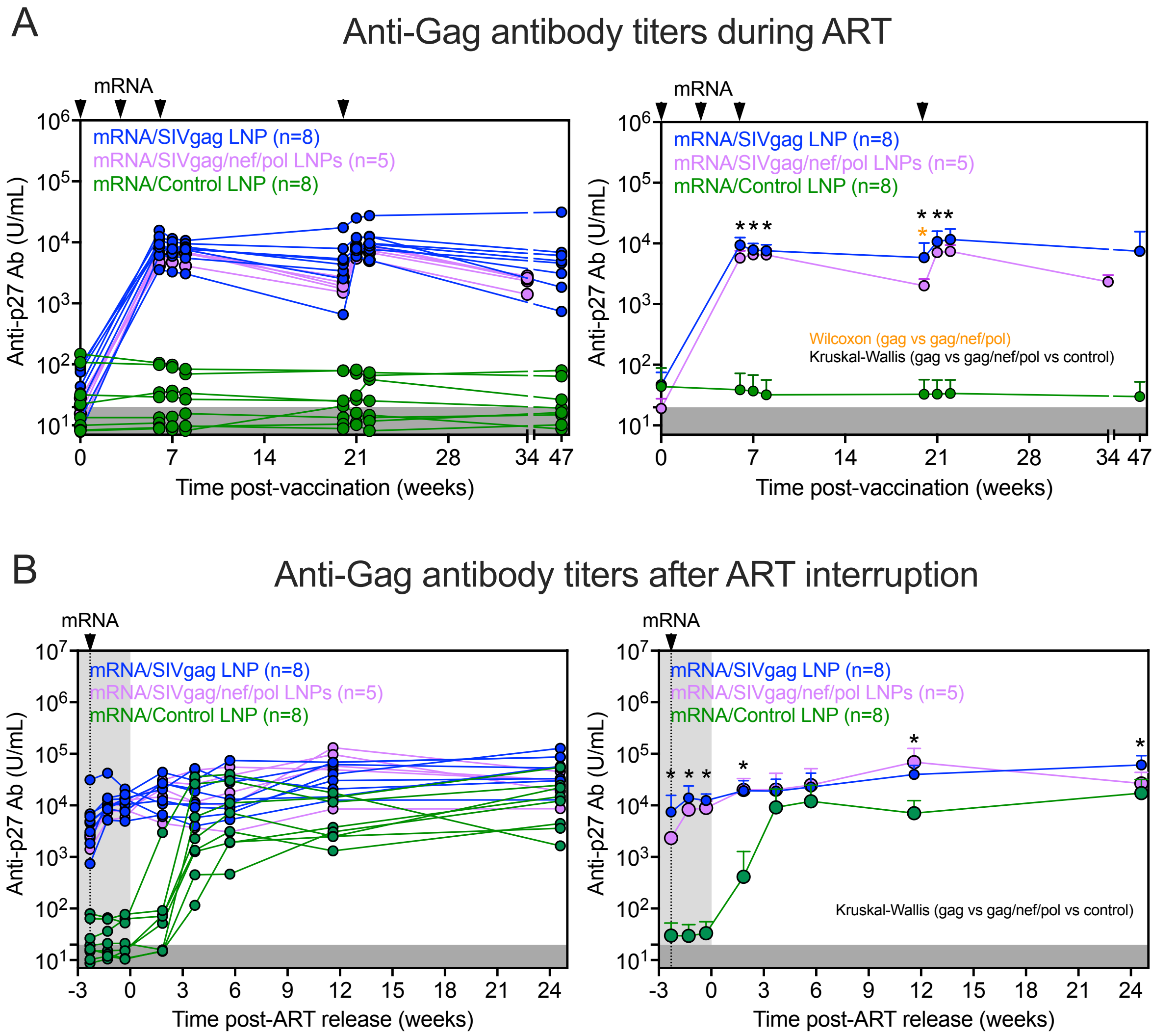

Supplemental Figure 10. Gag-specific antibody responses induced by vaccination. (A) Quantification of SIV Gag-specific antibody titers in plasma from SIVmac239M-infected RM on ART, vaccinated with mRNA/SIVgag, mRNA/SIVgag/nef/pol, or mRNA/control vectors. Individual antibody titers are shown in the left panel; group means (+ SEM) are shown in the right panel. (B) Quantification of SIV Gag-specific antibody titers in the same RMs following analytical treatment interruption (ATI). Individual titers are shown in the left panel; group means (+ SEM) are shown in the right panel. Statistical significance between groups was determined by Kruskal-Wallis test. When applicable, post-hoc two-sample Wilcoxon rank-sum (WRS) test was performed between pairs of treatment groups, without adjustment for multiple comparisons; p-values ≤0.05 are indicated.

Animal ID	Sex	Treatment Group	Age at time of SIV infection (years)	Viral challenge strain (5000 IU)	Peak plasma viral loads (Log SIV RNA copies/ml)	ART START (days post-SIV infection)	Time on ART (days)	Known protective MHC-1 allele
C1	Male	mRNA/SIVnef	6.1	SIVmac239M	6.6	9	492	
C2	Male	mRNA/SIVnef	3.3	SIVmac239M	6.6	9	492	B*08
C3	Male	mRNA/SIVnef	4.1	SIVmac239M	6.8	9	492	
D1	Male	mRNA/SIVpol	4.2	SIVmac239M	7.6	9	492	
D2	Male	mRNA/SIVpol	4.3	SIVmac239M	7.3	9	492	
D3	Male	mRNA/SIVpol	4.3	SIVmac239M	6.9	9	492	

Supplemental Table 3. Characteristics of RM in the study evaluating the immunogenicity of mRNA/SIVnef and mRNA/SIVpol vaccines. The table shows the sex, age at the time of SIV infection, challenge virus, peak plasma viral loads, duration of of ART relative to SIV infection and known protective MHC-1 alleles.

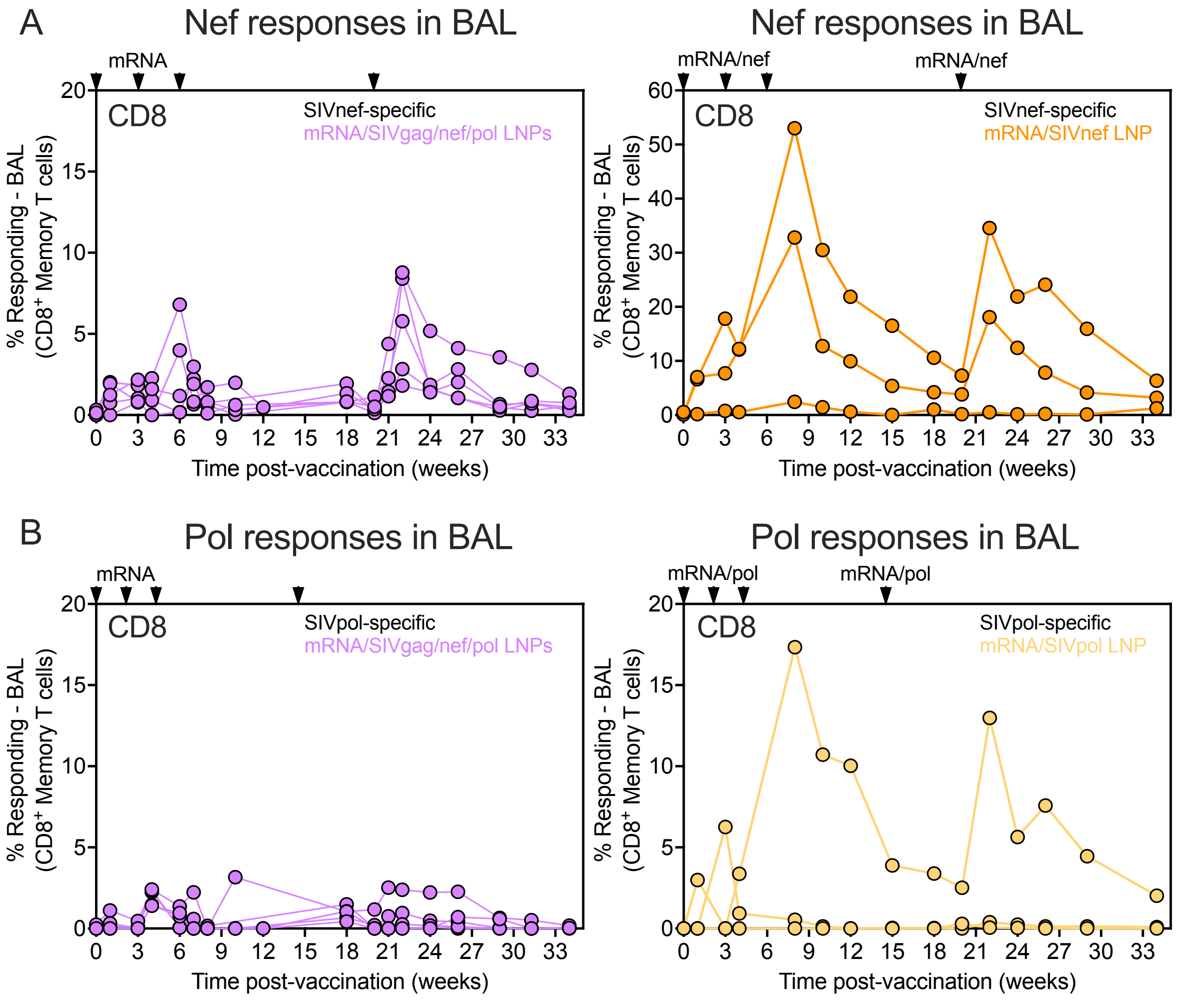

Supplemental Figure 11. Nef- and Pol-specific CD8$^+$ T cell responses induced by mRNA/SIVnef and mRNA/SIVpol vaccines. (**A**) SIV CD8$^+$ T cell responses to Nef in BAL of SIVmac239M infected RM that received mRNA/SIVgag/nef/pol (left panel) or mRNA/SIVnef alone (right panel) during ART. (**B**) SIV CD8$^+$ T cell responses to Pol in BAL of SIVmac239M infected RM on ART following vaccination with either mRNA/SIVgag/nef/pol (left panel) and mRNA/SIVnef alone (right panel).

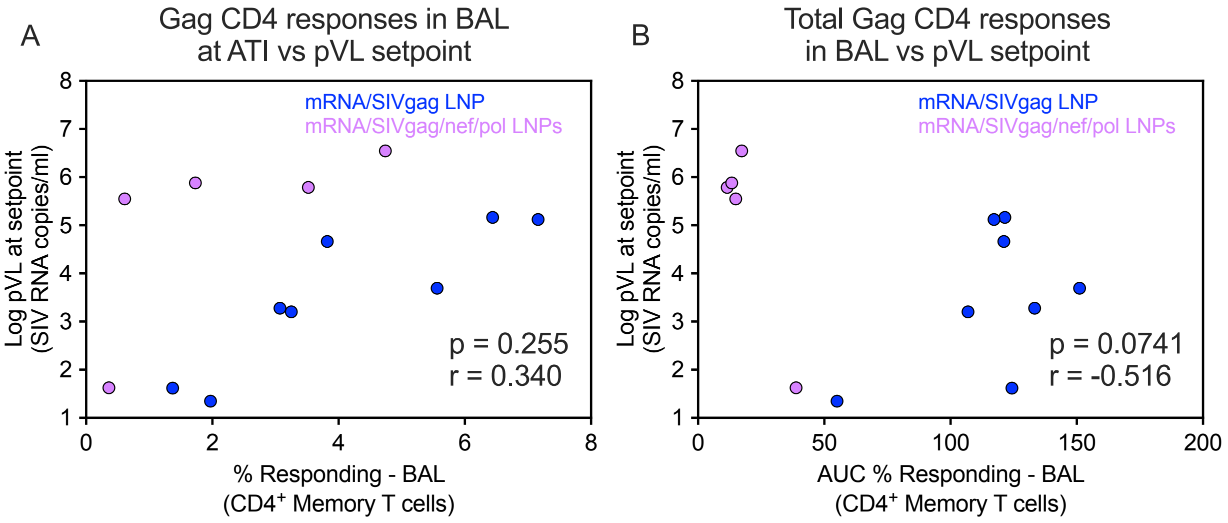

Supplemental Figure 12. No significant correlation between Gag-specific CD4^{+} T cell responses and post-ART viral load set points. (**A**) Scatterplots of Gag-specific CD4^{+} T cell responses in BAL at the time of ATI versus PVL 24 weeks post-ATI. (**B**) Scatterplots of total Gag-specific CD4^{+} T cell responses in BAL (measured from 0 to 34 weeks after the first vaccination) versus PVL 24 weeks post-ATI. Spearman rank correlation coefficients (r) with unadjusted p-values are shown for scatterplots to assess associations between immune responses and viral loads.
